# Hyperbaric Oxygen Boosts Antitumor Efficacy of Copper-Diethyldithiocarbamate Nanoparticles against Pancreatic Ductal Adenocarcinoma by Regulating Cancer Stem Cell Metabolism

**DOI:** 10.34133/research.0335

**Published:** 2024-03-11

**Authors:** Chen Xiao, Jiayuan Li, Ao Hua, Xing Wang, Shiyou Li, Zheng Li, Chen Xu, Zhijie Zhang, Xiangliang Yang, Zifu Li

**Affiliations:** ^1^ Department of Nanomedicine and Biopharmaceuticals, College of Life Science and Technology, Huazhong University of Science and Technology, Wuhan 430074, P. R. China.; ^2^ National Engineering Research Center for Nanomedicine, Huazhong University of Science and Technology, Wuhan 430074, P. R. China.; ^3^ Key Laboratory of Molecular Biophysics of Ministry of Education, Huazhong University of Science and Technology, Wuhan 430074, P. R. China.; ^4^ Hubei Key Laboratory of Bioinorganic Chemistry and Materia Medical, Huazhong University of Science and Technology, Wuhan 430074, P. R. China.; ^5^ Hubei Engineering Research Center for Biomaterials and Medical Protective Materials, Huazhong University of Science and Technology, Wuhan 430074, P. R. China.; ^6^ Hubei Bioinformatics and Molecular Imaging Key Laboratory, Huazhong University of Science and Technology, Wuhan 430074, P. R. China.

## Abstract

Cuproptosis-based cancer nanomedicine has received widespread attention recently. However, cuproptosis nanomedicine against pancreatic ductal adenocarcinoma (PDAC) is severely limited by cancer stem cells (CSCs), which reside in the hypoxic stroma and adopt glycolysis metabolism accordingly to resist cuproptosis-induced mitochondria damage. Here, we leverage hyperbaric oxygen (HBO) to regulate CSC metabolism by overcoming tumor hypoxia and to augment CSC elimination efficacy of polydopamine and hydroxyethyl starch stabilized copper-diethyldithiocarbamate nanoparticles (CuET@PH NPs). Mechanistically, while HBO and CuET@PH NPs inhibit glycolysis and oxidative phosphorylation, respectively, the combination of HBO and CuET@PH NPs potently suppresses energy metabolism of CSCs, thereby achieving robust tumor inhibition of PDAC and elongating mice survival importantly. This study reveals novel insights into the effects of cuproptosis nanomedicine on PDAC CSC metabolism and suggests that the combination of HBO with cuproptosis nanomedicine holds significant clinical translation potential for PDAC patients.

## Introduction

Although big coup has been achieved on treatments of several types of cancers during the last three decades, little progress has been made on the management of pancreatic cancers [1]. In particular, the 5-year survival rate of pancreatic ductal adenocarcinoma (PDAC) is still lower than 10% [2–6]. One of the reasons for this dismal efficacy is that PDAC has a special stroma with overactivated cancer-associated fibroblasts (CAFs) as well as excessive extracellular matrix (ECM) [4,7–13], which not only serve as physical barriers inhibiting drug delivery but also more importantly establish a unique niche to foster PDAC cancer stem cells (CSCs) [4,7,13–18]. CAFs and ECM, on the one hand, directly support CSCs survival and maintenance by secreting growth factors or cytokines and stimulating stem cell signaling pathways [19–23], on the other hand, indirectly sustain stemness by compressing intratumor blood vessels and inducing hypoxia to activate hypoxia-inducible factor–1α (HIF-1α) and the downstream signaling pathways [24]. While CSCs are notorious for drug resistance and are blamed as the main culprit for tumor recurrence and distant metastasis [25–28], CSCs’ energy metabolism represents a vulnerable target [29–31]. However, PDAC CSCs exhibit metabolic plasticity, i.e., both glycolysis and oxidative phosphorylation (OXPHOS) have been utilized by PDAC CSCs for energy supply [32–35]. It is therefore essential to suppress both glycolysis and OXPHOS of PDAC CSCs for potent cancer therapy.

Cuproptosis emerges as a novel form of cell death dependent on copper [36–38], which targets lipoylated tricarboxylic acid (TCA) proteins to induce oligomerization of dihydrolipoamide *S*-acetyltransferase (DLAT) and dihydrolipoamide *S*-succinyltransferase (DLST) and disrupt OXPHOS metabolism. Whereas disulfiram has been evaluated against numerous types of solid malignancies, including PDAC, in more than 20 clinical trials [37–39], the key metabolite copper-diethyldithiocarbamate (CuET), which is responsible for disulfiram-induced cell death, has an extremely low aqueous solubility (far less than 1 μg/l) [40]. As a result, multifarious nanodrug delivery systems are designed to enhance CuET aqueous solubility for tumor targeting delivery of copper and cuproptosis cancer nanomedicine has become a research hotspot [41–45]. Although extensive studies have utilized disulfiram and/or CuET to eliminate CSCs of various types of cancers [46–58], the impact of cuproptosis on PDAC CSCs metabolism is largely uncharted thus far. Even less explored is the influence of hypoxia on cuproptosis nanomedicine for PDAC treatment, not to mention the mechanism by which cuproptosis nanomedicine eradicates PDAC CSCs. As cuproptosis mainly involves TCA proteins in mitochondria while PDAC CSCs normally locate within dense and hypoxic stroma and exhibit metabolism plasticity, addressing these issues bears critical implications for treating PDAC with cuproptosis-based therapy in clinical settings.

**Fig. 1.**
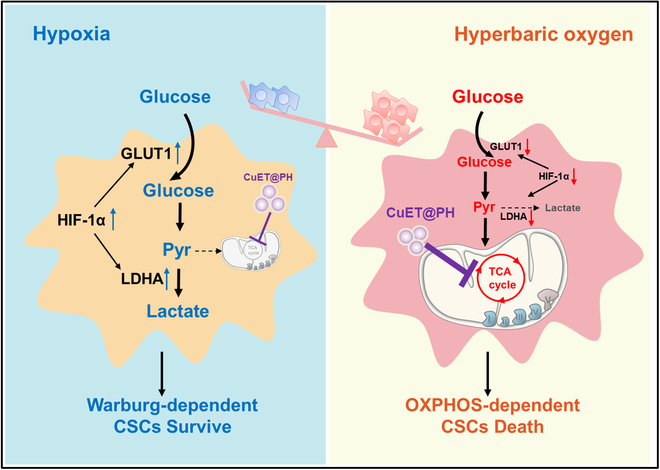
Schematic illustration of boosting CuET@PH NPs with HBO. Under pathological hypoxia, PDAC CSCs survive via Warburg effect, i.e., undergoing glycolysis and CuET@PH exhibiting marginal effect on CSCs. HBO overcomes tumor hypoxia and reprograms PDAC CSCs metabolism into an OXPHOS-dependent manner, where CuET@PH disrupts TCA cycle and eliminates PDAC CSCs.

Our group has proposed rational design of nanotherapeutics based on the five features principle for potent elimination of CSCs [59]. We developed a new strategy to remove CSCs by modulating the mechanical microenvironment of solid tumors [60–62] and constructed a variety of nanomedicines to disrupt redox homeostasis of CSCs [63–66]. We also used hyperbaric oxygen (HBO) to help commercialized nanomedicines exterminate CSCs by depleting CAFs and ECM within PDAC [67–68]. We further regulated CSCs metabolism by small molecular drugs and uncovered the mechanisms by which these drugs erase CSCs [69–71]. In a very recent study, we surprisingly found that clinical colloidal plasma volume expander hydroxyethyl starch (HES) stabilized copper-diethyldithiocarbamate nanoparticles (CuET@HES NPs) at an extraordinarily high efficiency and demonstrated that the as-prepared CuET@HES NPs killed CSCs of triple-negative breast cancer and hepatocellular carcinoma in not only tumor-bearing mice but also fresh tumor tissues derived from liver cancer patients [46]. However, CuET@HES NPs exhibited poor pharmacokinetic properties; upon systemic administration, they accumulated at tumor tissues at a very low efficiency and hardly suppressed tumor growth. In other words, CuET@HES NPs could only be administered locally, which severely limits their clinical applications. In this work, we modify CuET@HES NPs with polydopamine (PDA) and thiol-modified HES to afford CuET@PH NPs for systemic administration and further augment the antitumor efficacy of CuET@PH NPs against PDAC with HBO. We hypothesize that hypoxia helps PDAC CSCs survive by Warburg effect and renders PDAC CSCs insensitive toward CuET@PH NPs, while HBO reprograms PDAC CSCs metabolism from glycolysis to OXPHOS and thereby sensitizes PDAC CSCs vulnerable to CuET@PH NPs (Fig. 1). We test these hypotheses by using targeted metabolomics as well as biological tools and study the mechanism by which CuET@PH NPs eradicate PDAC CSCs by digging into CSC metabolism. Finally, we evaluate the efficacy and safety of CuET@PH NPs and HBO combination therapy in an orthotopic PDAC tumor model with a focus on cuproptosis-induced CSCs elimination.

## Results

**Fig. 2.**
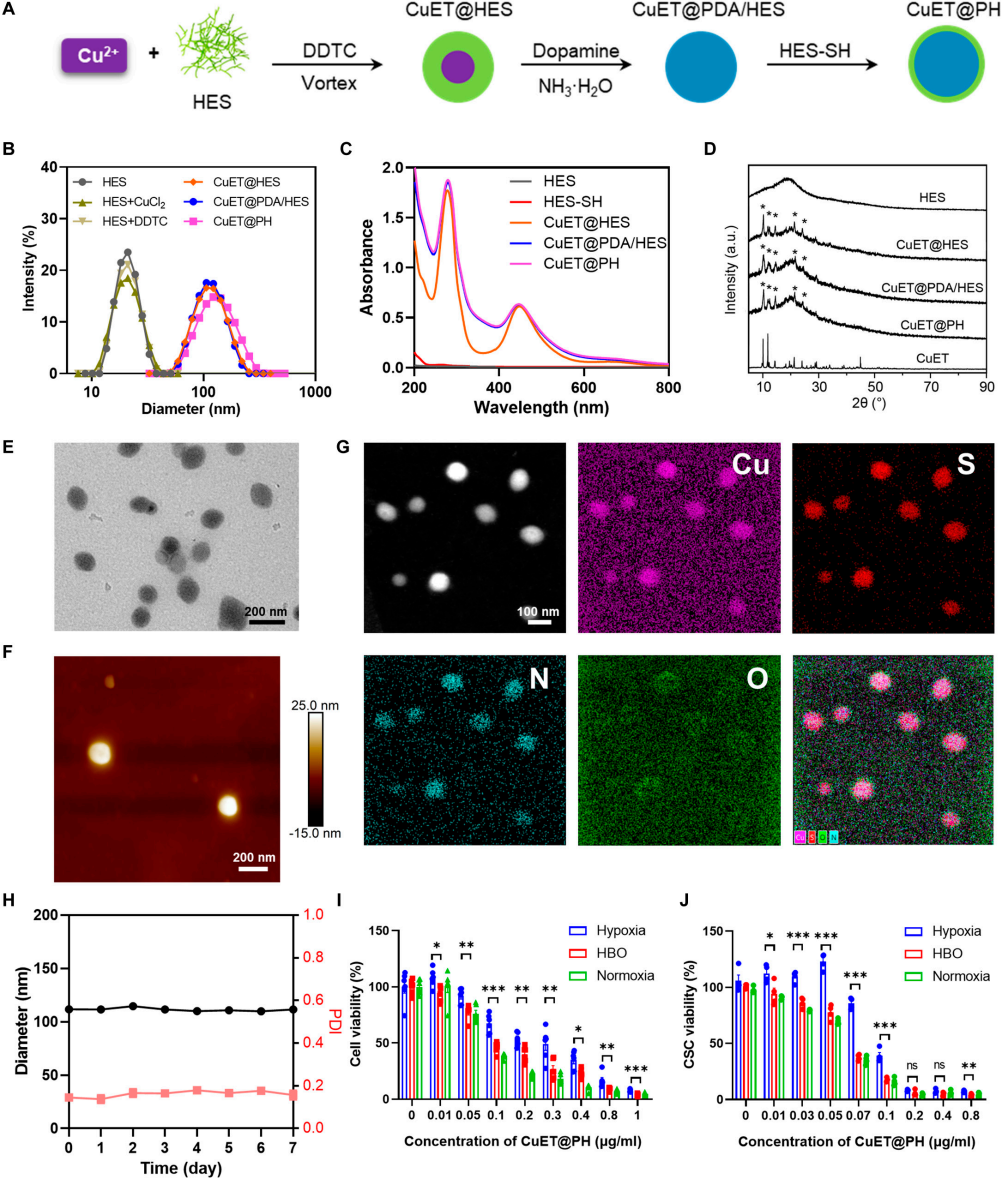
CuET@PH NPs characterization and cell killing ability. (A) Schematic illustrating preparation processes of CuET@PH NPs. (B) Size distributions of CuET@PH and intermediates during CuET@PH preparation. (C) UV-vis spectra of CuET@PH and intermediates during CuET@PH preparation. (D) XRD spectra of HES, CuET, CuET@HES, CuET@PDA/HES, and CuET@PH. TEM (E) and AFM (F) characterizing the morphology of CuET@PH. (G) HR-TEM characterizing the element distribution of CuET@PH NPs (scale bar: 100 nm). (H) Diameter and PDI of CuET@PH in 10% FBS solution during 7 days. Panc02 cells (I) and CSCs (J) killing assay by CuET@PH under hypoxia, HBO, and normoxia (mean ± SEM, *n* = 4). Statistical significance was calculated by *t* test. *P < 0.05, **P < 0.01, ***P < 0.001; ns, not significant.

The fabrication of CuET@PH NPs is schematically illustrated in Fig. 2A. First, CuET@HES NPs are prepared with the method reported before [46]. Then, to improve colloidal stability for systemic administration, CuET@HES NPs are coated with PDA and thiol-functionalized hydroxyl ethyl starch (HES-SH; Figs. S1 and S2) to afford CuET@PH NPs [72,73]. Consistent with previous studies [74,75], HES 200/0.5 with a molecular weight of 200 kDa and hydroxy ethyl substitution degree of 50% exhibits a colloidal diameter around 20 nm in dynamic laser light scattering (DLS) measurement. Figure 2B also demonstrates that CuET@PH NPs have a hydrodynamic diameter around 122 nm, which is slightly bigger than CuET@HES NPs, suggesting that PDA and HES-SH have been successfully decorated on the surface of CuET@HES NPs. The zeta potential of CuET@PH NPs was measured to be −10.8 mV, which is more negative than that of free CuET (21.2 mV), CuET@HES (−0.2 mV), and CuET@PDA/HES (−8.1 mV). These data further confirm the successful modification of PDA and HES-SH on CuET@PH NPs. Nonetheless, the functionalization with PDA and HES-SH does not change the molecular and crystal structures of CuET, as manifested in ultraviolet-visible (UV-vis) absorbance (Fig. 2C) and X-ray diffraction (XRD) pattern (Fig. 2D). The advantage of coating CuET@HES NPs with two layers of PDA and HES-SH lies in that the as-prepared CuET@PH NPs possess the best lyophilization stability (Fig. S3). Inductively coupled plasma-optical emission spectrometer (ICP-OES) measurements reveal that the Cu content in CuET@PH NPs is 0.76%, indicating that the drug content of CuET in CuET@PH NPs is approximately 4.3%. In excellent agreement with DLS results, transmission electron microscopy (TEM) and atomic force microscopy (AFM) corroborate monodisperse and spherical CuET@PH NPs that are obtained, and exhibited a uniform diameter distribution with polymer dispersity index (PDI) around 0.145 (Fig. 2E and F). High-resolution TEM (HR-TEM) reveals that copper, sulfur, nitrogen, and oxygen elements are uniformly dispersed throughout each single NP (Fig. 2G), indicating that CuET homogeneously distributed within CuET@PH NPs. These results contrast with the previous observation that CuET formed a dense core; CuET@HES NPs exhibited a dense core loose shell structure [46]. It is highly possible that CuET involves dopamine polymerization process during which the dense core loosens and CuET molecules and nanocrystals distribute evenly across CuET@PH NPs. Based on the x-ray photoelectron spectroscopy (XPS) results presented in Fig. S4, it has been demonstrated that HES stabilizes CuET nanocrystals through copper–oxygen coordination interactions, resulting in the formation of CuET@HES NPs [46]. Subsequent modification with PDA further reduces the binding energy of copper in both CuET@PDA/HES and CuET@PH NPs, compared to CuET@HES NPs, as evidenced in Fig. S4. This indicates that PDA can interact with the copper elements in CuET, thereby augmenting the stability of CuET. Figure 2H illustrates that CuET@PH NPs are stable in 10% fetal bovine serum (FBS) solution for at least 7 days. Considering that the outer layer of CuET@PH NPs is functionalized with HES, this result is reasonable, as HES is highly hydrophilic and can withstand protein adsorption [71–75]. Therefore, CuET@PH NPs have the potential for systemic administration.

Before in vivo explorations, we first evaluate the cytotoxicity of CuET@PH NPs on Panc02 cancer cells and CSCs in vitro. CSCs were derived from Panc02 cancer cells using the classic method by combining ultra-low attachment plate with CSC culture medium [67–70]. Figure 2I and J shows the viability of Panc02 cancer cells and CSCs, respectively, in varied concentrations of CuET@PH under hypoxia, HBO, and normoxia. Consistent conclusions could be drawn. Minimum half-maximal inhibitory concentration (IC_50_) values are obtained in normoxia for both cancer cells and CSCs (Tables S1 and S2). Hypoxia significantly abrogates killing capacity of CuET@PH. For Panc02 cancer cells, IC_50_ increases from 0.077 μg/ml in normoxia to 0.299 μg/ml under hypoxia; for CSCs, IC_50_ raises from 0.062 to 0.086 μg/ml, confirming our hypothesis that pathological hypoxia helps PDAC CSCs survive. Encouragingly, the cytotoxicity of CuET@PH NPs against both Panc02 cancer cells and CSCs could be recovered by HBO. In detail, the IC_50_ value is 0.134 and 0.064 μg/ml for Panc02 cancer cells and CSCs, respectively, under HBO. Note that CuET@PH NPs are more potent in killing Panc02 CSCs than cancer cells under normoxia and HBO, indicating that CuET@PH NPs have the advantages to eliminate PDAC CSCs. Similar conclusions have also been drawn with small drugs inducing abundant reactive oxygen species (ROS) [64,69,70]. This comparison suggests that hypoxia exerts a more significant impact on the cytotoxicity of CuET@PH NPs against Panc02 cancer cells than that toward CSCs and supports our hypothesis that HBO could almost fully recover the potency of CuET@PH NPs in eliminating PDAC CSCs. Furthermore, The Cancer Genome Atlas (TCGA) results support that CSCs are strongly and negatively correlated with PDAC patients’ progression-free survival (Fig. S5). Therefore, we next focus on investigating the impact of CuET@PH NPs on PDAC CSCs under hypoxia and HBO to uncover the mechanism by which CuET@PH NPs eradicate Panc02 CSCs.

**Fig. 3.**
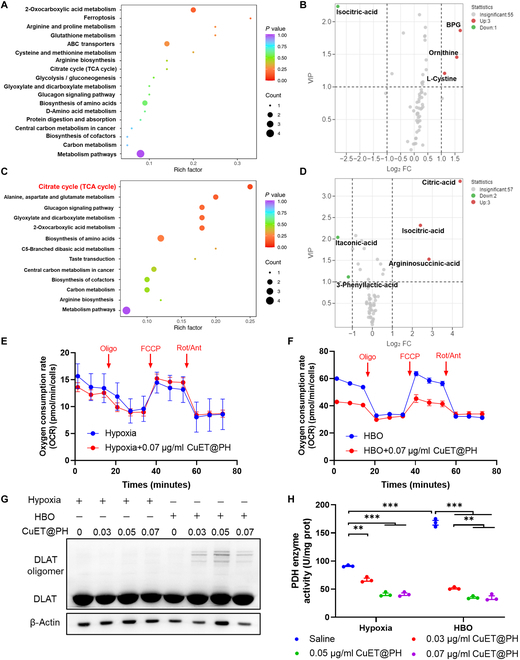
HBO helps CuET@PH NPs interrupt TCA cycle in Panc02 CSCs. KEGG-enriched pathway analysis based on metabolite differences between hypoxia and hypoxia combined with 0.03 μg/ml CuET@PH (A) as well as between HBO and HBO combined with 0.03 μg/ml CuET@PH (C). Up- and down-regulation of metabolites in volcano plots of hypoxia versus hypoxia combined with CuET@PH (B) and HBO versus HBO combined with CuET@PH (D), based on targeted metabolomic analysis (*n* = 6, FC ≥ 2, VIP ≥ 1). OCR of Panc02 CSCs in hypoxia (E) and HBO (F) after 24 hours of 0.07 μg/ml CuET@PH treatment (Oligo, oligomycin; FCCP, carbonylcyano-4-trifluoromethoxyphenylhydrazone; Rot/Ant, rotenone/antimycin A) (mean ± SEM, *n* = 4). (G) WB analysis of Panc02 CSCs treated with different concentrations of CuET@PH in hypoxia or HBO for 24 h. (H) PDH enzyme activity of Panc02 CSCs treated with different concentrations of CuET@PH in hypoxia or HBO for 24 h. Statistical significance was calculated by *t* test. **P < 0.01, ***P < 0.001.

We assume that hypoxia diminishes cytotoxicity of CuET@PH NPs by programming Panc02 CSCs metabolism. For this purpose, targeted metabolomics have been utilized to detect the metabolic effects of CuET@PH NPs on Panc02 CSCs under hypoxia and HBO conditions. Kyoto Encyclopedia of Genes and Genomes (KEGG)-enriched pathway analysis based on metabolite differences between CuET@PH NPs plus hypoxia and hypoxia is presented in Fig. 3A. From this perspective, it seems that CuET@PH NPs exert little influence on Panc02 CSC metabolism. However, 1,3-bisphospholycerate (BPG), ornithine, and l-cystine are up-regulated by 3.2401-, 2.9645-, and 2.1722-fold, respectively, whereas isocitric acid is down-regulated by 64.5161 times (Fig. 3B and Table S3), indicating that CuET@PH NPs stimulate Panc02 CSCs glycolysis and inhibit OXPHOS in hypoxia. In stark contrast, CuET@PH NPs mainly affect citrate cycle (TCA cycle) of Panc02 CSCs under HBO, as shown in Fig. 3C. Figure 3D and Table S4 further reveal that citric acid, isocitric acid, and argininosuccinic acid are remarkably up-regulated between CuET@PH NPs plus HBO and HBO. Of note, citric acid has been increased by 206.3007 times. To directly study the impacts of CuET@PH NPs on Panc02 CSC metabolism, oxygen consumption rate (OCR) is measured with seahorse. Figure 3E indicates that similar results are obtained under hypoxia with and without the introduction of CuET@PH NPs, while Fig. S6 demonstrates that no significant difference can be detected in terms of relative OCR under baseline and stressed conditions. These results suggest that under hypoxia CuET@PH NPs exert marginal effect on Panc02 CSC mitochondria respiration. Contrastingly, CuET@PH NPs significantly suppress Panc02 CSC mitochondria respiration under HBO (Fig. 3F). In detail, Fig. S7 reveals that CuET@PH NPs repress Panc02 CSCs basal respiration by 28.3% and maximal respiration by 47.3%, respectively, supporting that CuET@PH NPs potently inhibit spare respiratory capacity of Panc02 CSCs. These results suggest that CuET@PH NPs target on Panc02 CSCs mitochondria. Because the main active pharmaceutical ingredient in CuET@PH NPs is CuET, which was recently revealed to target lipoylated TCA cycle proteins and induce DLAT and DLST oligomers [36], we checked the expressions of DLAT by Western blot (WB) with varied concentrations of CuET@PH NPs under hypoxia and HBO. Figure 3G displays that DLAT oligomers are only formed under HBO, whereas no DLAT oligomer is formed under hypoxia under at all tested concentrations of CuET@PH NPs. Since DLAT is a key component of pyruvate dehydrogenase (PDH) complex, the oligomerization of DLAT will affect PDH activity. To this end, we tested PDH activity. Figure 3H exhibits that PDH activity is constrained to a higher extent under HBO than under hypoxia in the presence of CuET@PH NPs. For example, at a concentration of 0.03 μg/ml CuET@PH NPs, PDH activity is decreased by 26.7% in hypoxia and 69.3% in HBO, respectively. Collectively, these results corroborate that HBO helps CuET@PH NPs disrupt TCA cycle in Panc02 CSCs, whereas hypoxia impairs the activity of CuET@PH NPs, providing plausible explanations for hypoxia-deteriorated and HBO-augmented cytotoxicity of CuET@PH NPs against Panc02 CSCs (Fig. 2J). Nonetheless, the effects of hypoxia or HBO on Panc02 CSCs are not uncovered, although clear differences in terms of basal respiration and PDH activity have been noted between hypoxia and HBO (Fig. 3E, F, and H).

**Fig. 4.**
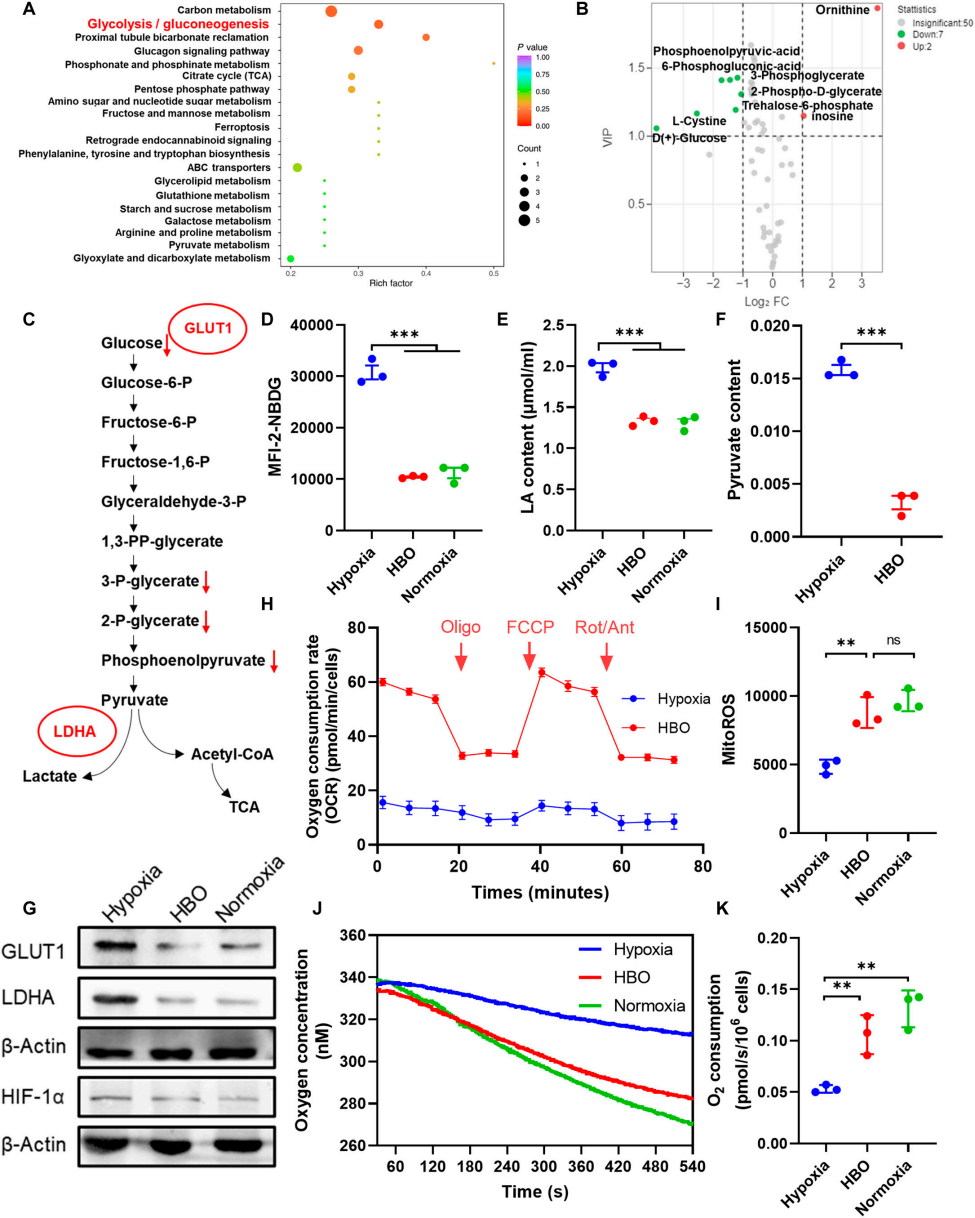
HBO inhibits glycolysis and enhances OXPHOS in Panc02 CSCs. (A) KEGG-enriched pathway analysis based on metabolite differences between hypoxia and HBO. (B) Up- and down-regulation of metabolites in volcano plots of hypoxia versus HBO based on targeted metabolomic analysis (*n* = 6, FC ≥ 2, VIP ≥ 1). (C) Decreased metabolites in the HBO group compared to the hypoxia group in the glycolytic pathway. Levels of 2-NBDG uptake (D), intracellular LA (E), and intracellular pyruvate (F) of Panc02 CSCs under hypoxia, HBO, and normoxia by flow cytometry (mean ± SEM, *n* = 3). (G) WB analysis of Panc02 CSCs treated with hypoxia, HBO, and normoxia for 24 h. (H) OCR of Panc02 CSCs under hypoxia and HBO (mean ± SEM, *n* = 4). (I) Levels of intracellular MitoROS under hypoxia, HBO, and normoxia by flow cytometry (mean ± SEM, *n* = 3). (J) Measurement of oxygen consumption of Panc02 CSCs under different treatment using a Clark oxygen electrode. (K) Quantification of Panc02 CSCs OCRs (mean ± SEM, *n* = 3). Statistical significance was calculated by *t* test. *P < 0.05, **P < 0.01, ***P < 0.001.

Next, we sought to study the influence of hypoxia and HBO on Panc02 CSCs metabolism. By using targeted metabolomics, we have detected the metabolite differences between HBO and hypoxia. KEGG-enriched pathway analysis in Fig. 4A indicates that carbon metabolism, including glycolysis and gluconeogenesis, is regulated in Panc02 CSCs by comparing HBO with hypoxia, while Fig. 4B displays that ornithine and inosine are significantly up-regulated but d-glucose, 3-phosphoglyceric acid, 2-phosphoglycerate, phosphoenolpyruvate, 6-phosphogluconic acid, and l-cystine are remarkably down-regulated. Table S5 summarizes the results and shows that ornithine has been increased by 56.1953-fold, whereas d-glucose has been reduced by 15.015 times. These results highlight that glycolysis in Panc02 CSCs is conspicuously repressed by HBO, as illustrated in Fig. 4C. To validate the conclusions drawn from targeted metabolomics, we further quantified several key metabolites in Panc02 CSCs under hypoxia and HBO. Instead of tracking glucose, we used 2-deoxy-2-[(7-nitro-2,1,3-benzoxadiazol-4-yl) amino]-d-glucose (2-NBDG) to study glucose uptake by Panc02 CSCs. Figure 4D reveals that HBO suppresses 2-NBDG uptake by 66.1% relative to hypoxia. Figure 4E and F also indicates that HBO inhibits the production of lactate (LA) by 32.8% and pyruvate by 79.4%, respectively. Furthermore, Fig. 4G demonstrates that HBO constrains glycolysis in Panc02 CSCs by overcoming hypoxia, which up-regulates the expression of glucose transporter type 1 (GLUT1) and lactate dehydrogenase A (LDHA) and facilitates glycolysis metabolism. The gene expressions of HIF-1α, GLUT1, and LDHA are also decreased by HBO (Fig. S8). Crucially, the gene expressions of GLUT1 and LDHA are strongly and negatively correlated with PDAC patients’ overall survival and progression-free survival (Figs. S9 and S10), implying that HBO might provide survival benefits for PDAC patients. These results also suggest that HBO switches Panc02 CSCs metabolism from glycolysis to OXPHOS. To substantiate these hypotheses, seahorse was used. Figure 4H exhibits the measured OCR values of Panc02 CSCs under hypoxia and HBO; 3.82- and 4.43-fold enhancements are noted in basal respiration and maximal respiration after HBO treatments (Fig. S11). Meanwhile, HBO significantly down-

regulated the proton efflux rate compared with hypoxia (Fig. S12). Accordingly, mitochondrial ROS (MitoROS) and adenosine triphosphate (ATP) are increased by 1.82- and 2.06-fold after HBO treatments (Fig. 4I and Fig. S13). Oxygen consumption by Panc02 CSCs is also directly measured with Clark oxygen electrode, as shown in Fig. 4J and K, and consistent conclusions can be drawn. Together, these results corroborate that HBO disrupts tumor hypoxia to inhibit glycolysis and promotes OXPHOS in Panc02 CSCs, rendering Panc02 CSCs susceptible to CuET@PH NPs-induced cuproptosis.

**Fig. 5.**
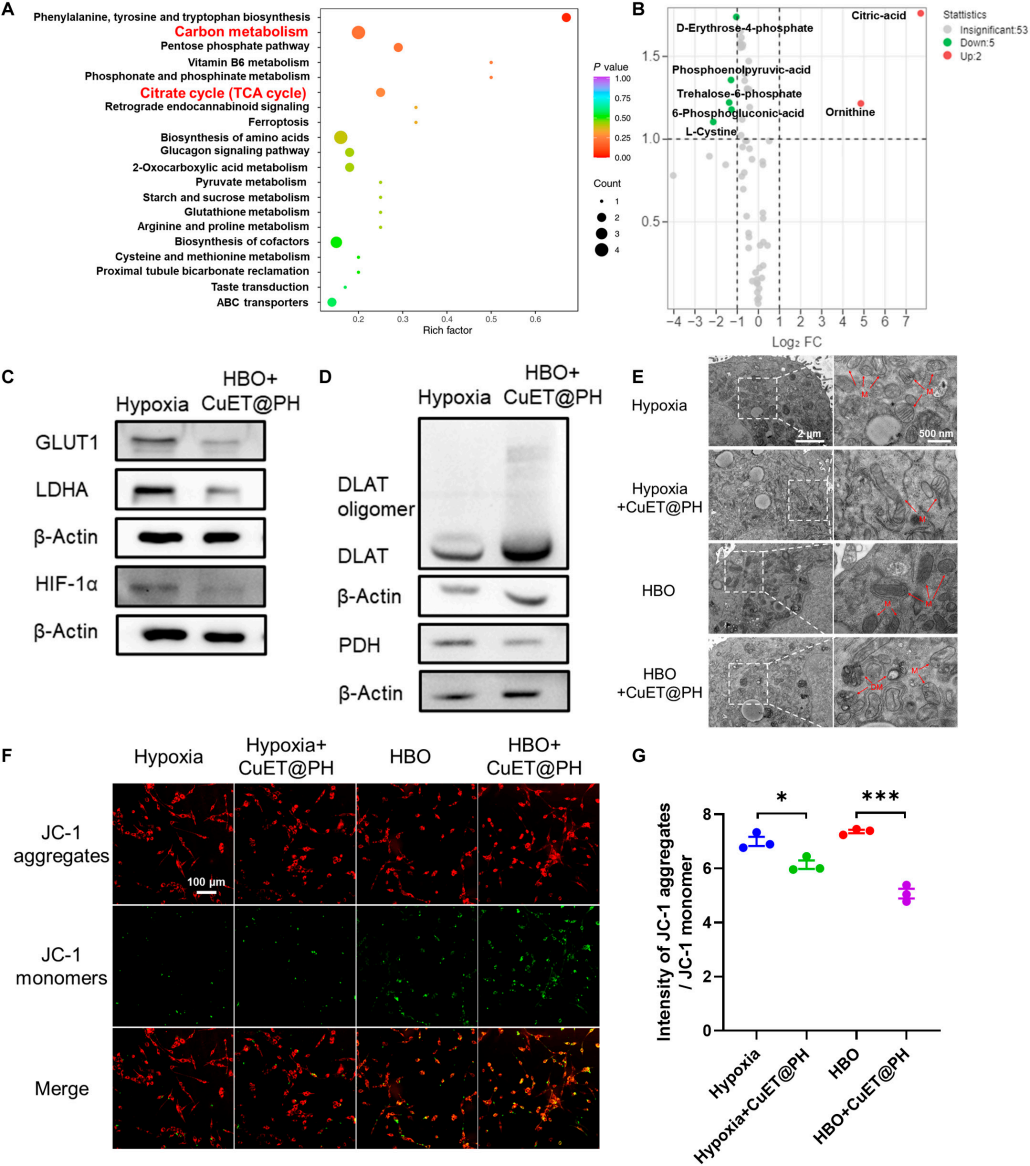
HBO synergizes with CuET@PH NPs to inhibit energy metabolism and disrupt mitochondria in Panc02 CSCs. (A) KEGG-enriched pathway analysis based on metabolite differences between hypoxia and HBO combined with 0.03 μg/ml CuET@PH. (B) Up- and down-regulation of metabolites in volcano plots of hypoxia versus HBO combined with 0.03 μg/ml CuET@PH based on targeted metabolomic analysis (*n* = 6, FC ≥ 2, VIP ≥ 1). (C) WB analysis of Panc02 CSCs treated with hypoxia and HBO combined with 0.05 μg/ml CuET@PH. (D) WB analysis of Panc02 CSCs treated with hypoxia and HBO combined with 0.05 μg/ml CuET@PH. (E) Mitochondrial structure imaging under different treatments by Bio-TEM. The concentration of CuET@PH was 0.05 μg/ml. (F) Confocal imaging of Panc02 CSCs mitochondrial membrane potential using JC-1 fluorescent probe under different treatments. The concentration of CuET@PH was 0.05 μg/ml (scale bar: 100 μm). (G) Quantification of the ratio between JC-1 aggregates and JC-1 monomer by flow cytometry. The concentration of CuET@PH NPs was 0.05 μg/ml (mean ± SEM, *n* = 3). Statistical significance was calculated by *t* test. *P < 0.05, ***P < 0.001.

To reveal the mechanism by which HBO helps CuET@PH NPs eliminate PDAC CSCs, we compared metabolite differences between HBO plus CuET@PH NP group and hypoxia group. KEGG-enriched pathway analysis in Fig. 5A indicates that carbon metabolism, in particular TCA cycle, is modulated. In detail, citric acid and ornithine are significantly up-regulated by 206.3007- and 28.904-fold, whereas phosphoenolpyruvate, 2-phospho-d-glycerate, and l-cystine are down-regulated by the combination treatment (Fig. 5B and Table S6). Consistent with Fig. 4, these results support the conclusion that HBO plus CuET@PH NPs combination treatments suppress glycolysis and interrupt OXPHOS in Panc02 CSCs. This conclusion is also supported by WB analysis; as illustrated in Fig. 5C, the protein expressions of HIF-1α, GLUT1, and LDHA are suppressed by the combination treatment. Besides, the gene expressions are also reduced by the combination treatment (Fig. S14). Intriguingly, 6-phosphogluconic acid and d-erythrose-4-phosphate are also significantly down-regulated by the combination treatments, signifying that the pentose phosphate pathway (PPP) is disrupted. Since DLAT oligomers were triggered (Fig. 5D) and MitoROS were uplifted by the combination of HBO and CuET@PH NPs, we further examined mitochondria structure and function of Panc02 CSCs after different treatments. Figure 5E and Fig. S15 show that damaged mitochondria are only evident in HBO and CuET@PH NPs combination treatments, which also exhibit the lowest mitochondrial membrane potential (Fig. 5F and G). Therefore, Panc02 CSCs stemness and proliferation are suppressed the most by HBO and CuET@PH NPs combination treatments in in vitro fibrin gel assay (Fig. S16) [68–70]. Compared with colony spheroids formed in hypoxia, HBO solely decreases colony number by 37.2% and colony diameter by 30.9%. Presumably, HBO diminishes Panc02 CSC stemness and proliferation by switching CSCs metabolism from glycolysis to OXPHOS and enhancing MitoROS generation. Under hypoxia, CuET@PH NPs inhibit stemness and proliferation by 67.3% and 56.1%, respectively; however, the suppression effects are enhanced to 80.6% and 70.1% by HBO. Collectively, Figs. 3 to 5 not only reveal the mechanism by which hypoxia abrogates cuproptosis-induced cell death but also more importantly delineate how HBO facilitates CuET@PH NPs to eradicate Panc02 CSCs in vitro.

**Fig. 6.**
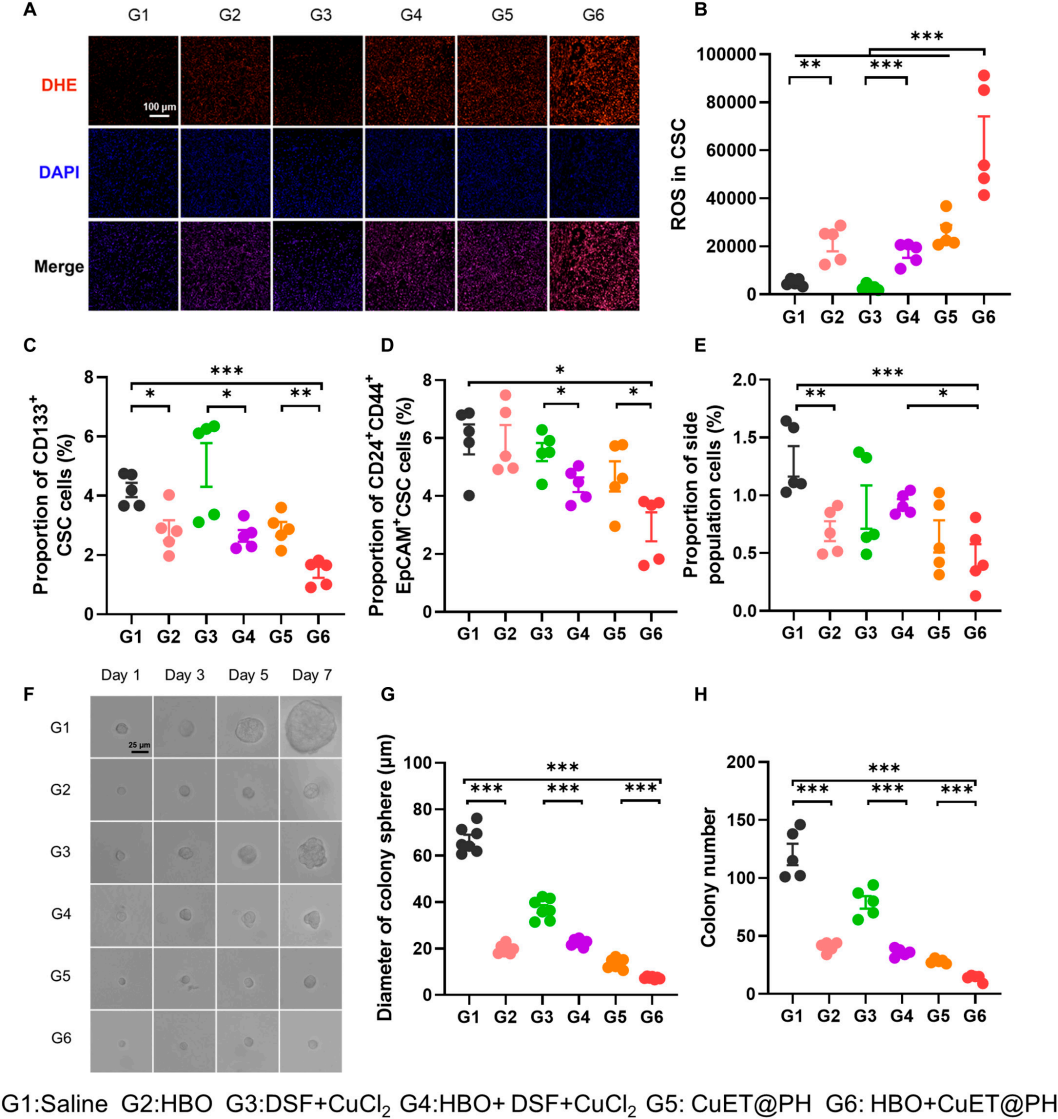
HBO helps CuET@PH NPs eliminate Panc02 CSCs in vivo. (A) ROS immunofluorescence of tumor tissues after different treatments (scale bar: 100 μm). (B) Levels of CSCs ROS in tumor tissues of different treatments by flow cytometry (mean ± SEM, *n* = 5). Proportion of CD133^+^ CSCs (C), CD24^+^CD44^+^ CSCs (D), and side-population cells (E) in tumor tissues of different treatments by flow cytometry (mean ± SEM, *n* = 5). (F) 3D fibrin gel experiments with cancer cells derived from tumor tissues of different groups (scale bar: 25 μm). (G) Diameter of colony sphere on day 7 of different groups (mean ± SEM, *n* = 7). (H) Colony number on day 7 of different groups (mean ± SEM, *n* = 5). Statistical significance was calculated by *t* test. *P < 0.05, **P < 0.01, ***P < 0.001.

Next, we sought to investigate the effects of HBO and CuET@PH NPs on Panc02 CSCs in vivo. First, we studied biodistribution of CuET@PH NPs in an orthotopic Panc02 tumor model (Fig. S17). In vivo and ex vivo imaging corroborate that HBO enhances CuET@PH NPs tumor targeting delivery; semiquantification of ex vivo imaging exhibits that HBO promotes CuET@PH NPs tumor targeting efficiency by 2.58 times. We further dissected Panc02 tumor tissues into single-cell suspension and measured the internalized CuET@PH NPs by Panc02 tumor cells. Flow cytometry analysis manifests that HBO augments endocytosis by 2.24-fold. Our previous study documented that HBO potently modulated aberrant tumor mechanics in Panc02 tumors by suppressing CAFs and depleting ECM [68]. As such, the physical barriers for CuET@PH delivery have been removed by HBO. Therefore, these results are expected and in excellent agreement with previous studies [63,67,68,76]. Because CuET@PH NPs-induced cuproptosis mainly targets TCA cycle and amplifies MitoROS for CSC elimination, we then evaluated oxidative status after various treatments. Figure 6A illustrates that combination treatments of HBO and CuET@PH NPs (G6) trigger the highest oxidative stress in Panc02 tumor tissues; semiquantification of immunofluorescence in Fig. S18 signifies that combination treatments increase ROS by 7.4 times relative to that of saline (G1). The levels of ROS within Panc02 CSCs were further probed with flow cytometry by dissecting Panc02 tumor tissues into single-cell suspension. Several interesting observations could be noted in Fig. 6B. Consistent with the in vitro results illustrated in Fig. 4, HBO alone (G2) could elevate CSC ROS by 4.23-fold relative to saline (G1) in tumor tissues. Whereas free disulfiram (DSF) and CuCl_2_ (G3) could hardly enhance ROS levels, the introduction of HBO (G4) conspicuously lifts ROS levels by 3.44 times, confirming that HBO enhances ROS generation. CuET@PH NPs increase CSCs ROS by 5.19 times. The combination of HBO and CuET@PH NPs (G6) boosts CSCs intracellular ROS to the highest level among all groups, around 12.79-fold of saline. As a result of upsurged ROS, the least number of CSCs is measured in G6 in terms of CD133^+^, CD24^+^CD44^+^, and side-population identified Panc02 CSCs (Fig. 6C to E). Specifically, combination treatments have suppressed CD133^+^, CD24^+^CD44^+^, and side-population CSCs by 66.3%, 50.9%, and 64.3% to those of saline. The CSC function was further investigated by culturing cells, which are derived from Panc02 tumor tissues after different treatments, into three-dimensional (3D) soft fibrin gels [68–70]. Figure 6F displays Panc02 tumor spheroids on days 1, 3, 5, and 7. Clearly, the smallest tumor spheroid is observed in G6, which has a mean diameter of 7.4 μm, whereas the mean diameter is around 66.92 μm in the saline group (Fig. 6G), indicating that Panc02 CSCs proliferation is remarkably constrained. Similarly, Panc02 CSCs stemness is also repressed by the combination treatments of HBO and CuET@PH NPs. As shown in Fig. 6H, the colony spheroid number has been declined from 120 in G1 to 13 in G6. Another interesting observation is that HBO alone (G2) significantly curbs the functions of Panc02 CSCs, which is consistent with in vitro results. Together, Fig. 6 corroborates that HBO helps CuET@PH NPs eliminate Panc02 CSCs in vivo by diminishing the number of CSCs and restraining CSCs functions.

**Fig. 7.**
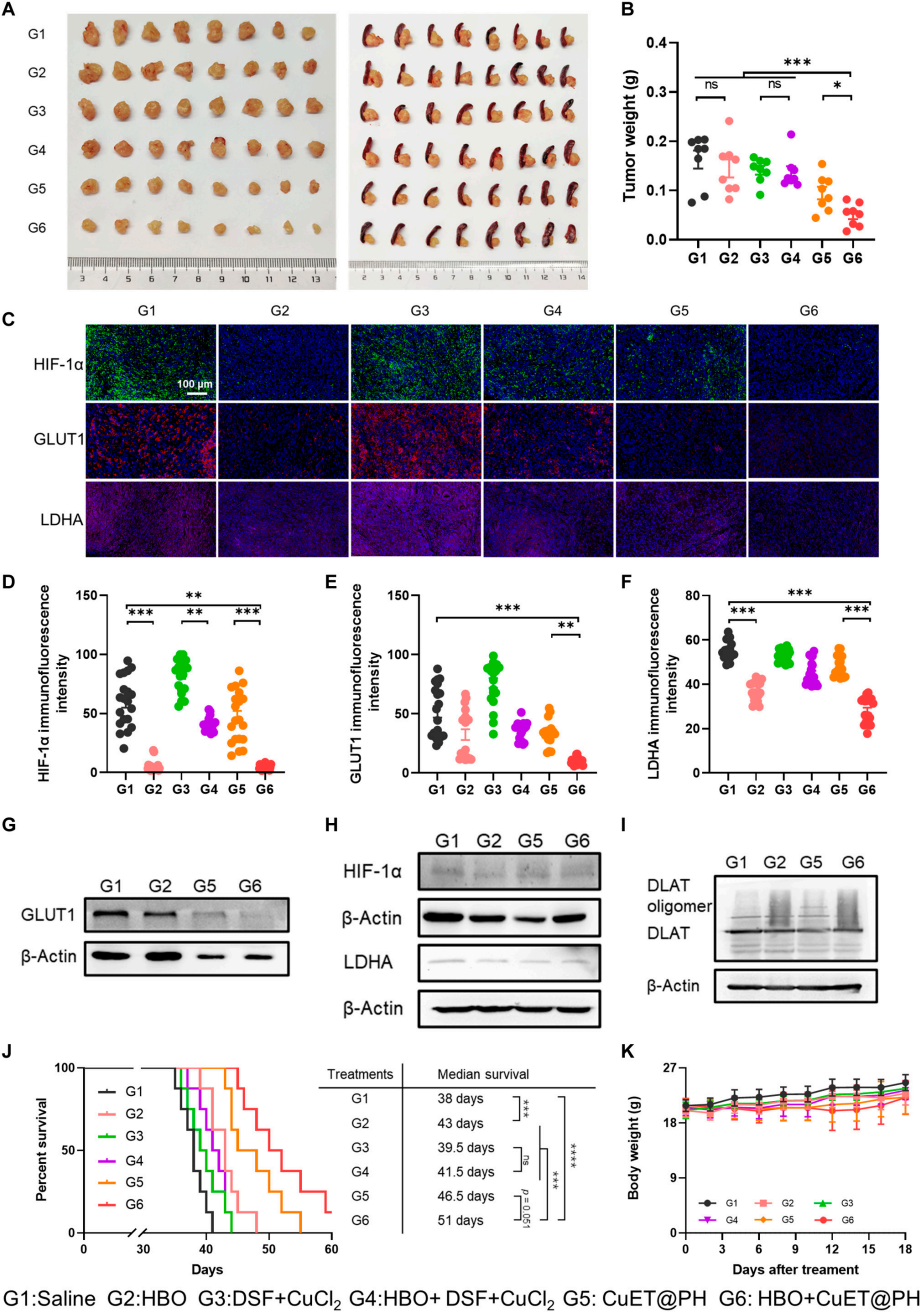
HBO boosts antitumor efficacy of CuET@PH NPs against Panc02 tumors and prolongs mice survival. (A) Images of excised tumors of different groups. (B) Excised tumor weight of different groups (mean ± SEM, *n* = 8). (C) HIF-1α, GLUT1, and LDHA immunofluorescence sections of tumor tissues with different groups (scale bar: 100 μm). Immunofluorescence intensity of HIF-1α (D), GLUT1 (E), and LDHA (F) in tumor tissues from different groups (mean ± SEM, *n* = 20). (G to I) WB analysis of tumor tissues with different groups. (J) Survival curve of different groups (*n* = 8) as analyzed by the log-rank (Mantel–Cox) test. (K) Body weight of mice of different groups (mean ± SEM, *n* = 8). Statistical significance was calculated by *t* test. *P < 0.05, **P < 0.01, ***P < 0.001.

Finally, we tested the antitumor effect of combination treatments of HBO and CuET@PH NPs in orthotopic Panc02 tumors. The images and weights of excised tumors after different treatments are presented in Fig. 7A and B, respectively. The combination treatments achieve the highest tumor inhibition rate (TIR) of 69%, followed by CuET@PH NPs (G5) with a TIR of 42%, G4 (16%), and G3 (13%). Note that although HBO alone suppressed Panc02 CSCs to a decent extent (Fig. 6), no significant difference is observed between G2 and G1 in tumor weights. Similar conclusion can be drawn by comparing G4 with G3, emphasizing that HBO selectively boosts antitumor effects of nanomedicines rather than small molecular drugs [63,67,68,76]. It was also worth noticing that DSF + CuCl_2_ did not show any antitumor effect in orthotopic Panc02 tumors. These findings align with our previously reported data, which indicated that DSF + CuCl_2_ did not show any antitumor efficacy in either 4T1 subcutaneous tumor model or H22 ascites tumor model [45]. These dismal antitumor outcomes could be ascribed to poor circulation abilities of DSF and a low accumulation of CuET at tumor sites. Subsequently, the tumor inhibition mechanisms are studied in orthotopic Panc02 tumors. Consistent with in vitro results, combination treatments of HBO and CuET@PH NPs down-regulate the protein and gene expressions of GLUT1 and LDHA by overcoming tumor hypoxia (Fig. 7C to G and Fig. S19). These results confirm that HBO and CuET@PH NPs restrain glycolysis in Panc02 tumor tissues. Furthermore, the combination treatments induce robust cuproptosis effects in vivo, as exemplified by oligomerizations of DLAT (Fig. 7H and Fig. S20). As a result, tumors in G6 display the least proliferation (Fig. S21) and the highest necrosis (Fig. S22) among all groups. Accordingly, mice in G6 accomplish the longest survival, as illustrated in Fig. 7J. It is interesting to point out that HBO alone (G2) elongates mice survival significantly than saline (G1). The median survival in G2 prolongs 5 days relative to G1. This effect could be ascribed to the fact that HBO suppresses CSCs-mediated metastasis [63,67,68]. However, HBO-induced survival elongation is not detected by comparing G4 with G3, reinforcing the conclusion that HBO preferentially augments nanomedicines than small-molecule agents, such as DSF and CuCl_2_. No abnormality is observed in mice body weight (Fig. 7K), hematoxylin-eosin (H&E) staining of major organs (Fig. S23), and blood routine as well as blood biochemistry (Fig. S24), indicating that these treatments all have negligible side effects. These results together substantiate that HBO augments antitumor efficacy of CuET@PH NPs for PDAC treatments and prolongs mice survival.

## Discussion

In this study, we propose a novel combination therapy of HBO and CuET@PH NPs to reprogram PDAC CSCs metabolism. The rationale lies in that HBO switches CSCs metabolism from glycolysis to OXPHOS and CuET@PH NPs target TCA cycle to trigger cuproptosis cell death. Our study suggests the combination treatments potently disrupt PDAC CSC energy metabolism to eliminate CSCs, inhibit tumor growth, and prolong mice survival.

Because the aberrant mechanical microenvironment consists of overactivated CAFs and excessive ECM, PDAC CSCs are normally located in a hypoxic region distant from blood vessels. Accordingly, glycolytic metabolism is adopted by PDAC CSCs; glycolysis inhibitors, e.g., 3-bromopyruvate, could sensitize PDAC CSCs toward gemcitabine [34]. Nonetheless, Viale et al. [32] demonstrated that PDAC CSCs, which were responsible for tumor recurrence, relied on OXPHOS metabolism and Sancho et al. [33] and Zhao et al. [77] revealed that the metabolic phenotypes were determined by the balance between MYC and PGC-1α. Furthermore, PDAC CSCs metabolic phenotypes were correlated with organ-specific colonization; liver metastasis exhibited glycolysis, while lung metastasis showed OXPHOS [35]. These studies emphasize that PDAC CSCs have metabolic plasticity and require that both glycolysis and OXPHOS should be inhibited for potent CSCs elimination. For this purpose, we leverage HBO to force PDAC CSCs switch from glycolysis to OXPHOS and target TCA cycle with CuET@PH NPs for cuproptosis cell death [36]. Compared with other drugs inhibiting glycolysis, HBO has three incomparable advantages. First, HBO utilizes high-pressured pure oxygen to disrupt tumor hypoxia. With a smaller molecular weight than most glycolysis inhibitors, HBO can penetrate as deep as 100 μm away from blood vessels [78,79]. Second, besides inhibiting glycolysis, HBO also significantly augments nanomedicine tumor targeting delivery efficiency by regulating the aberrant tumor mechanics [63,67,68,76]. Third, HBO has been widely used in clinical settings with more than 13 approved indications [78]. Therefore, the combination of HBO with cuproptosis nanomedicines holds great translational potential.

While cuproptosis cancer nanomedicine has attracted tremendous attention recently [41–58,80,81], the influence of hypoxia, a typical physical abnormality for most solid malignancies, on cuproptosis-induced cell death is seldom explored thus far [36]. In principle, hypoxia would be detrimental to cuproptosis cancer nanomedicine, as hypoxia would render cancer cells and more importantly CSCs resistant to oligomerizations of DLAT and DLST by reprogramming energy metabolism. Hypoxia-deteriorated cytotoxicity of CuET@PH NPs has been demonstrated in Fig. 2I and J, and the mechanisms by which hypoxia weakens CuET@PH NP-triggered cuproptosis have been revealed in Figs. 3 to 5. These results provide plausible explanations to account for the dismal outcomes of cuproptosis-based clinical trials [39,41]. Crucially, HBO boosts the effects of CuET@PH NPs by switching CSCs metabolism from glycolysis to OXPHOS (Figs. 3 to 7). As such, this study reveals a novel function of clinically used HBO, i.e., HBO is a potent glycolysis inhibitor. Therefore, the introduction of HBO is beneficial for cuproptosis cancer nanomedicine. For future cuproptosis cancer therapy clinical trials, a concomitant hypoxia-disrupting adjuvant therapy is critical and highly recommended.

Regarding PDAC CSCs metabolism, there are several interesting points worthy of further discussion. First, because copper targets lipoylated TCA proteins in mitochondria, oxidative stress is key for cuproptosis cell death. Under hypoxia, PDAC CSCs undergo glycolysis metabolism and MitoROS production is low. Even in the presence of CuET@PH NPs, mitochondria structure and function are not interrupted. HBO alone lifts the generation of MitoROS by switching PDAC CSCs metabolism from glycolysis to OXPHOS and therefore suppresses Panc02 CSCs both in vitro and in vivo. With the assistance of HBO, CuET@PH NPs boosts MitoROS generation and eliminate CSCs. Second, to counteract the damage induced by oxidative stress, CSCs elevate intrinsic antioxidant systems to up-regulate the expressions of reducing agents [82]. l-Cystine is up-regulated by 2.1722-fold in comparing hypoxia plus CuET@PH NPs with hypoxia (Fig. 3B and Table S3), which also explains why CuET@PH NPs induce limited mitochondria damage (Fig. 5F) and cytotoxicity (Fig. 2J) under hypoxia. In stark contrast, l-cystine is significantly down-regulated in comparing HBO with hypoxia (Fig. 4B and Table S5) as well as HBO plus CuET@PH NPs with hypoxia (Fig. 5B and Table S6). Similarly, PPP is repressed to a decent extent in these two comparisons. Since l-cystine is a critical resource for glutathione biosynthesis and PPP is essential for glutathione reduction [66], reduced l-cystine and blocked PPP are conducive to cuproptosis-triggered oxidative stress [83]. Third, two key metabolites, including ornithine (Figs. 3C, 4B, and 5B and Tables S3, S5, and S6) and argininosuccinic acid (Fig. 3D and Table S4), are constantly up-regulated, signifying that arginine metabolism is stimulated within PDAC CSCs in these conditions. It is understandable that Panc02 CSCs utilize other substances, such as glutamine, for metabolic compensation when TCA cycle in glucose metabolism is blocked [70,84]. To the best of our knowledge, this is the first report of cuproptosis nanomedicine promoting arginine metabolism. Future work could be carried out by combining cuproptosis nanomedicine with arginine metabolism inhibitors.

We acknowledge some limitations for the current study. First, although targeted metabolomics are used, metabolic flux analysis has not been leveraged in our study. Mechanistic studies on PDAC CSCs metabolism are insufficient. Second, several confirmatory biological experiments are performed in terms of cuproptosis cell death. The impacts of hypoxia and/or HBO on the mechanisms of cuproptosis nanomedicines await further studies. Third, the universality of the conclusions drawn from this study needs to be tested further, since only one type of PDAC tumor and single cuproptosis nanomedicine are studied.

## Conclusion

In summary, we leverage HBO, for the first time, to boost cuproptosis nanomedicine (CuET@PH NPs) for potent CSCs elimination and efficient inhibition of PDAC. This study has fourfold implications. First, we demonstrate that cuproptosis nanomedicine is hampered by hypoxia and reveal that PDAC CSCs survive via Warburg effect to withstand cuproptosis-induced mitochondria damage. Therefore, cuproptosis-based cancer therapy should be performed simultaneously with hypoxia disruption therapy. Second, we illustrate that HBO potently suppresses PDAC CSCs glycolysis by inhibiting HIF-1α to down-regulate the expressions of GLUT1 and LDHA proteins. As more than 20 clinical trials have been carried out by treating cancer patients with cuproptosis-based therapy, HBO might benefit these patients further. Third, considering that CSCs exhibit metabolism plasticity, this study highlights the significance of simultaneously inhibiting both glycolysis and OXPHOS for eliminating CSCs. Fourth, this study lays the foundation of treating PDAC patients with combination therapy of HBO and cuproptosis nanomedicine. It is of great significance to test the efficacy and safety of this combination therapy with prospective clinical trials in PDAC patients.

## Materials and Methods

### Materials

HES (200/0.5) was a gift from Wuhan HUST Life Science & Technology Co. Ltd. CuCl_2_, sodium diethyldithiocarbamate (DDTC), dopamine, and ammonia solution were purchased from Aladdin Reagent Inc. All agents were used directly without further purification. Cell Counting Kit-8 (C0038), Enhanced mitochondrial membrane potential assay kit with JC-1 (C2003S), Hoechst 33342 (C1022), and Enhanced ATP Assay Kit (S0027) were purchased from Beyotime (Shanghai, China). Anti-DLAT polyclonal antibody (K004409P) and anti-DLST polyclonal antibody (K004852P) were purchased from Solarbio (Beijing, China). GLUT1 rabbit monoclonal antibody (mAb) (73015S), LDHA rabbit mAb (3582 T), HIF-1α rabbit mAb (48085S), β-actin rabbit mAb (4970S), and MitoTracker Deep Red FM (8778S) were purchased from Cell Signaling Technology (Massachusetts, USA). 2-NBDG (186689-07-6) and verapamil (52-53-9) were purchased from MedChem Express (USA). Lactic Acid (LA) Content Assay Kit (BC2235) and Pyruvate (PA) Content Assay Kit (BC2205) were purchased from Solarbio (Beijing, China). MitoSOX Red Mitochondrial Superoxide Indicator (40778es50) was purchased from YEASEN (Shanghai, China). Allophycocyanin (APC) anti-mouse CD133 (141208), fluorescein (FITC) anti-mouse CD24 (101806), and PE/Cyanine7 anti-mouse CD44 (103030) were purchased from Dakewe (Shenzhen, China). HiScript III RT SuperMix for qPCR (+gDNA wiper) (R323-01) and ChamQ Universal SYBR qPCR Master Mix (Q711-02) were purchased from Vazyme (Nanjing, China). Seahorse XF Cell Mito Stress Test Kit (103015-100) was purchased from Agilent (California, USA). Pyruvate Dehydrogenase (PDH) Activity Assay Kit (BC0385) was purchased from Solarbio (Beijing, China).

### Cell culture and animals

The Panc02 cancer cell line was obtained from Beijing Beinachuanglian Biotechnology Research Institute, and the cells were cultured in Dulbecco’s modified Eagle’s medium (DMEM) high-glucose medium containing 10% FBS and 1% antibiotics (penicillin: 100 U/ml, streptomycin: 100 μg/ml). Panc02 CSCs were derived from Panc02 cancer cells with a method reported before [67]. Typically, Panc02 cancer cells were cultured in low adherence plates with stem cell medium, which was DMEM/F12 medium containing 4 mg/ml bovine serum albumin (BSA), 20 ng/ml basic fibroblast growth factor (FGF), 20 ng/ml epidermal growth factor (EGF), 4 μg/ml insulin, and B27 (1:50). Then, the cells were placed in a 37 °C, 1% O_2_ hypoxic incubator, and the medium was changed every 2 days. After three passages, Panc02 CSCs are obtained.

C57BL/6J mice (male) were purchased from Vital River Laboratory Animal Technology Co. Mice were housed in an animal facility with constant environmental conditions (room temperature 21 ± 1 °C, relative humidity 40 to 70%, 12 h light–dark cycle). All mice were allowed to eat and drink freely. All animal experiments were approved by the Animal Care and Use Committee of Tongji Medical College of Huazhong University of Science and Technology (Wuhan, China). The experimental protocol was approved by the Animal Ethics Committee of Huazhong University of Science and Technology. The animal ethical review project number is 2019S924.

### Synthetic process of HES-SH

HES-SH was synthesized with three steps as reported before [72]. First, HES (1.0 g) reacted with succinic anhydride (0.07 g) in 25 ml of dimethyl sulfoxide (DMSO) solution, with 4-dimethylaminopyridine (DMAP) (0.06 g) catalyzed for 24 h at 45 °C, to obtain carboxylated HES. Second, carboxylated HES (0.5 g) reacted with 2-(pyridyldithio) ethylamine hydrochloride (0.06 g) in 20 ml of aqueous solution with EDCI (0.15 g) and *N*-hydroxy succinimide (NHS) (0.05 g) for 48 h at 25 °C to obtain HES-PA. Finally, to produce HES-SH, HES-PA (0.3 g) was reduced by dithiothreitol (0.48 g) in 20 ml of DMSO for 24 h at 45 °C under N_2_ atmosphere. All products were further purified with dialysis (3500 Da, Biosharp) and freeze-dried after reactions. The successful synthesis of HES-SH was determined by ^1^H-NMR (nuclear magnetic resonance) and FT-IR (Fourier transform infrared) spectra with a 600-MHz NMR spectrometer and a Nicolet iS50R FT-IR instrument.

### Preparation of CuET@PH NPs

CuET@PH NPs were prepared with three steps. First, HES (10 mg) and CuCl_2_ (5.57 μmol) were mixed in 1 ml of aqueous solution, and then DDTC solution (11.4 μmol, 0.2 ml) was added to the mixture under vigorous vortex at 60 °C to obtain CuET@HES NPs [46]. Second, dopamine (5 mg) and ammonia solution (1 ml) were added to the above CuET@HES NP solution and reacted for 12 h at 25 °C. CuET@PDA/HES NPs were thus acquired and purified with dialysis (3500 Da, Biosharp). Finally, HES-SH (20 mg) was added to the above CuET@PDA/HES NP solution and reacted for 48 h at 25 °C. The product CuET@PH NP solution was purified with ultrafiltration (100 kDa) three times for further usage.

### Characterization of CuET@PH NPs

DLS and zeta potential data were collected with Zetasizer Nano ZS90. XRD and XPS spectra were measured by x’pert3 powder and AXIS SUPRA+. TEM (Hitachi HT7700), FTEM (Talos F200s), and AFM (Bruker MultiMode8) were used to characterize the morphology of CuET@PH NPs.

### Cell killing assay in Panc02 cells

The cells were placed into 96-well rigid plates with 8000 Panc02 cells per well and incubated at 37 °C in a 5% CO_2_ cell incubator. As cells attached to the wall, the supernatant was removed and 100 μl of CuET@PH NP solutions of different concentrations were added, and then the cells were treated under hypoxia, HBO, and normoxia, respectively. In the hypoxia group, the plates were placed in a hypoxic cell incubator at 37 °C, 1% O_2_ for 24 h. In the HBO group, the plates were also placed in a hypoxic cell incubator at 37 °C, 1% O_2_ for 12 h. Then, the cell plates were transferred to the HBO chamber (Weifang Huaxin DS14-01), with the pressure set at 2.5 atmospheres absolute (ATA), and incubated with pure oxygen for 2 h. After HBO treatment, the plates were put back to the hypoxic cell incubator for another 10-h incubation. In the normoxia group, the plates were placed in a 37 °C, 5% CO_2_ cell incubator for 24 h. In all three groups, cell survival rate was calculated using the CCK-8 method 24 h after drug administration.

### Cell killing assay of Panc02 CSCs

The Panc02 CSCs were placed into 96-well low-adherent cell plates with 5000 Panc02 CSCs per well, while CuET@PH NP solutions of varied concentrations were added at the same time, with a final volume of 100 μl per well, and then treated according to the groups of hypoxia, HBO, and normoxia, respectively. In the hypoxia group, the plates were placed in a hypoxic cell incubator at 37 °C, 1% O_2_ for 24 h. In the HBO group, the plates were also placed in a hypoxic cell incubator at 37 °C, 1% O_2_ for 12 h. Then, the cell plates were transferred to the HBO chamber, with the pressure set to 2.5 ATA, and incubated with pure oxygen for 2 h. After HBO treatment, the plates were put back to the hypoxic cell incubator for another 10-h incubation. In the normoxia group, the plates were placed in a 37 °C, 5% CO_2_ cell incubator for 24 h. In all three groups, cell survival rate was calculated using the CCK-8 method 24 h after drug administration.

### Targeted metabolomics to detect the metabolic effects of CuET@PH on Panc02 CSCs in hypoxia and HBO conditions

Based on liquid chromatography tandem mass spectrometry (LC-MS/MS) detection platform, the metabolites of Panc02 CSCs were detected after different treatments. The groups were hypoxia, hypoxia + CuET@PH, HBO, and HBO + CuET@PH, and there were six replicates in each group. The experimental procedure was the same as the cell killing assay in Panc02 CSCs, and the concentration of CuET@PH NPs was 0.03 μg/ml. The experiment was performed in Wuhan Metavir Biotechnology Co. The experiment included the following steps: Panc02 CSCs collection and processing, metabolite extraction, metabolite detection, data preprocessing, data quality control, metabolite identification annotation, and differential metabolite analysis. Differential metabolites were screened using a combination of VIP (variable important in projection) values and log_2_FC values. VIP values are variable weight values of the orthogonal partial least squares discrimination analysis (OPLS-DA) model variables, which can be used to measure the strength of influence and explanatory ability of the differences in the accumulation of each metabolite on the categorical discriminations of each group of samples. VIP ≥ 1 is differential metabolite screening criterion. Fold change (FC) values are used to calculate the fold difference in the expression of a metabolite between two groups based on the absolute quantification, and the screening criteria for differential metabolites are FC ≥ 2 for up-regulation or FC ≤ 0.5 for down-regulation.

### Seahorse OCR experiment

Seahorse (Seahorse XFe 96) was used to determine the OCR of Panc02 CSCs. Panc02 CSCs were pretreated, in which the experimental procedure was the same as that of Panc02 CSC killing assay, and the concentration of CuET@PH NPs was 0.07 μg/ml. With 3,000 cells per well, the pretreated Panc02 CSCs were placed in poly-d-lysine pre-coated Seahorse XFe 96 well plates, centrifuged at 200*g* for 1 min at room temperature, and incubated in a 37 °C cell culture incubator without supplemental CO_2_ for 25 to 30 min to ensure that Panc02 CSCs were fully adherent to plate wall. The probe plate was hydrated, and 20 μl of oligomycin (final concentration, 1.5 μM), 22 μl of carbonyl cyanide 4-(trifluoromethoxy) phenylhydrazone (FCCP) (final concentration, 1 μM), and 25 μl of rotenone/antimycin A (final concentration, 0.5 μM) were added into the wells of A, B, and C, respectively. Seahorse recorded OCR values of each group of Panc02 CSCs in real time.

### WB assay

WB was used to analyze the expression of GLUT1, LDHA, HIF-1α, and DLAT in Panc02 CSCs after different treatments. The procedure was as follows. (a) Protein extraction: First, Panc02 CSCs were treated with CuET@PH NPs (0.05 μg/ml) and oxygen environment according to different groups. Panc02 CSCs were collected, and an appropriate amount of radioimmunoprecipitation assay (RIAP) lysate and 1% phenylmethanesulfonyl fluoride (PMSF) were added to the cells. Protein concentration of the cells was determined by bicinchoninic acid assay (BCA) method, and 5 × loading buffer was added. Then, the proteins were boiled for 10 min at 100 °C to denature the proteins and stored at −20 °C. (b) Electrophoresis: 10% SDS-PAGE was used for the upper layer of separator gel according to the measured protein concentration for sampling. The protein separation process was performed under the voltage of 100 V, and electrophoresis was stopped until the electrophoresis indicator bromophenol blue had reached the bottom of the gel. (c) Membrane transfer: The electrophoresed gel was separated from the glass plate and transferred into the transfer box placed according to the sandwich model, and the activated polyvinylidene difluoride (PVDF) membrane was covered on the gel. The process of membrane transfer was performed under a constant current; the selected current was 240 mA. The time of membrane transfer was selected according to the molecular weight of the target protein. (d) Antibody incubation: The transferred PVDF membrane was blocked with 5% skimmed milk of tris-buffered saline with Tween 20 (TBST) for 1 h. The primary antibody was incubated overnight at 4 °C and then washed three times, and the secondary antibody was incubated at room temperature for 1 h and then washed three times. (e) Imaging: Using chemiluminescence imaging system XRS+ for imaging and analyzing the expression level of target proteins.

### PDH enzyme activity of Panc02 CSCs

The experiment was divided into eight groups: hypoxia, hypoxia + 0.03 μg/ml CuET@PH, hypoxia + 0.05 μg/ml CuET@PH, hypoxia + 0.07 μg/ml CuET@PH, HBO, HBO + 0.03 μg/ml CuET@PH, HBO + 0.05 μg/ml CuET@PH, and HBO + 0.07 μg/ml CuET@PH. Panc02 CSCs were treated with different concentrations of CuET@PH NPs and oxygen environment according to different groups, in which the procedure was the same as that of Panc02 CSC killing assay. After cells were collected, PDH Activity Assay Kit was used and the test method was described in the instruction manual of the kit.

### 2-NBDG uptake assay

Panc02 CSCs were placed in low-adhesion six-well plates at 2 × 10^5^ cells per well, and the experiment was divided into three groups: hypoxia, HBO, and normoxia. In the hypoxia group, the low-adherent cell plates were placed into a 37 °C, 1% O_2_ incubator for 24 h. In the HBO group, the low-adherent cell plates were placed into the 37 °C, 1% O_2_ incubator for 12 h. The plates were then transferred to the hyperbaric chamber and incubated with pure oxygen for 2 h at 2.5 ATA, and then returned to the hypoxic incubator for another 10 h. In the normoxia group, the low-adhesion cell plates were placed in the cell culture incubator at 37 °C, 5% CO_2_ for 24 h. Then, 2-NBDG was added into the CSC culture medium of each group at a final concentration of 50 μM and incubated for 1 h. After washing three times with phosphate-buffered saline (PBS), the intensity of 2-NBDG in the Panc02 CSCs of each group was determined using flow cytometry (Cytoflex S).

### Lactate content assay

The lactate content assay included three groups: hypoxia, HBO, and normoxia. The cell pretreatment method was the same as that of 2-NBDG uptake assay. After the cells were collected, Lactic Acid (LA) Content Assay Kit was used, and the test method was described in the instruction manual of the kit.

### Pyruvate content assay

The pyruvate content assay included two groups: hypoxia and HBO. The cell pretreatment method was the same as that of 2-NBDG uptake assay. After the collection of cells, the Pyruvate (PA) Content Assay Kit was used, and the test method was described in the instruction manual of the kit.

### MitoROS level assay

MitoROS level assay included three groups: hypoxia, HBO, and normoxia. The cell treatment method was the same as that of 2-NBDG uptake assay. MitoSOX Red was added to the culture medium of each group at a final concentration of 5 μM and incubated for 30 min. After washing three times with PBS, the fluorescence intensity of MitoSOX Red in Panc02 CSCs of each group was measured by flow cytometry.

### Oxygen consumption determination by oximetric microelectrode

Oxygen concentration in the culture medium was determined using an oxygen microelectrode (Unisense) to indirectly calculate OCR of Panc02 CSCs. The experiment included three groups: hypoxia, HBO, and normoxia. The cell treatment method was the same as that used of 2-NBDG uptake experiment. The cells were collected by centrifugation, resuspended with 1 ml of Hanks’ balanced salt solution, and liquid-sealed using vegetable oil on the surface of the solution; the oxygen concentration in the medium was measured using a Clark oxygen electrode, with the data recorded at 1-s interval, and the duration of the measurement was 2 min.

### Changes in mitochondrial membrane potential

This experiment included four groups: hypoxia, hypoxia + CuET@PH, HBO, and HBO + CuET@PH. Panc02 CSCs were placed into low-adhesion six-well plates at 2 × 10^5^ cells per well, and hypoxia + CuET@PH and HBO + CuET@PH groups were added to the CuET@PH solution at the same time as the cells were added so that the final drug concentration was 0.05 μg/ml. Four sets of low-adhesion plates were placed at a 37 °C cell incubator with 1% O_2_. The hypoxia and hypoxia + CuET@PH groups were incubated in the hypoxic cell incubator for 24 h. The HBO and HBO + CuET@PH groups were incubated in the hypoxic cell incubator for 12 h and then transferred to an HBO chamber with a set pressure of 2.5 ATA and pure oxygen for 2 h. Cells after HBO treatment continued to incubate in the hypoxic cell incubator for 10 h. Groups of cells were collected, counted, and put into a poly-d-lysine pre-packaged 20-mm confocal dish at a volume of 1 × 10^5^ cells per dish. After CSCs adhered to the wall, aspirated the supernatant, added 1 ml of JC-1 staining solution, and incubated at 37 °C for 20 min. CSCs were washed twice with JC-1 staining buffer. Observation was performed with laser scanning confocal microscopy (FV3000).

### Bio-TEM of Panc02 CSCs

Bio-TEM was used to characterize the effects of drug treatment on the mitochondrial structure of Panc02 CSCs in the presence of hypoxia or HBO. The experimental procedures were divided into the following steps. (a) Preparation of cell samples and fixation: Panc02 CSCs were placed into low-adhesion six-well plates at 2 × 10^5^ cells per well, and the drug group added CuET@PH solution at the same time as adding the cells to make the final drug concentration of 0.05 μg/ml. The four groups of low-adhesion plates were placed for incubation in a hypoxic incubator at 37 °C, 1% O_2_. The hypoxia and hypoxia + CuET@PH groups were incubated in the hypoxic incubator for 24 h. The HBO and HBO + CuET@PH groups were incubated in the hypoxic incubator for 12 h and then transferred to a hyperbaric chamber, with the pressure set at 2.5 ATA and pure oxygen incubation for 2 h. The incubation was continued in the hypoxic incubator for another 10 h after HBO treatment. Cells of each group were collected and washed twice with PBS. After that, 2.5% glutaraldehyde was added for fixation overnight. (b) After fixation: 2.5% glutaraldehyde-fixed Panc02 CSCs were rinsed with 0.1 M PBS (pH 7.4) three times for 15 min each time and then fixed with 1% osmium acid for 2 h. The cells were then rinsed again with 0.1 M PBS (pH 7.4) three times for 15 min each time. (c) Dehydration: The samples were dehydrated by 30%, 50%, 70%, 80%, 85%, 90%, 95%, and 100% (twice) alcohol upstream for 20 min each time. (d) Osmosis: The osmotic agent was acetone:epoxy resin (2:1), acetone:epoxy resin (1:1), and epoxy resin, in order, in a 37 °C incubator for 12 h each time. (e) Embedding: The permeated samples were put into the capsule or embedding plate, added with the embedding agent epoxy, and polymerized for 48 h in a 60 °C incubator. (f) Sectioning: The thickness of section was 80 to 100 nm. (g) Double staining and observation: Uranium-lead double staining (2% uranium acetate saturated aqueous solution, lead citrate, room temperature staining for 15 min): The sections were dried at room temperature overnight, and the mitochondrial structure was characterized by TEM.

### In vivo pharmacodynamic studies, safety evaluations, and survival experiments

Pharmacodynamic studies were performed using the orthotopic Panc02 tumor model. The mice were 6-week male C57BL/6J mice with a weight of around 20 g.

Orthotopic Panc02 tumor model: 150 μl of 1% pentobarbital sodium solution was injected intraperitoneally into the mice. After the mice were anaesthetized, the abdominal tissues of the mice were incised using a scalpel, and 50 μl of PBS suspension containing 1 × 10^6^ Panc02 cells was injected into the pancreas of the mice, which was then sutured. When the tumors grew to day 7, the mice were randomly divided into six groups: saline, HBO, DSF + CuCl_2_, HBO + DSF + CuCl_2_, CuET@PH, and HBO + CuET@PH. CuET@PH was administered at a dose of 4 mg/kg, and DSF + CuCl_2_ was administered at a molar amount equal to that of CuET@PH and was administered by tail vein injection. The frequency of administration was every 2 days for a total of six doses. In the group with HBO, on the day of drug administration, the drug was first injected into the tail vein, and then the mice were placed in the HBO chamber, with the pressure set at 1.5 ATA, and treated with pure oxygen for 2 h. A portion of the mice was sacrificed at the end of the treatment; at the same time, the tumors were stripped, fixed, dehydrated, embedded, and sectioned. The necrotic area of the tumors in each group was investigated by H&E staining, while cell proliferation of the tumors tissues in each group was observed by using Ki67. HIF-1α, GLUT1, LDHA, and DLAT were used to quantify the expression of proteins in each tumor tissue, and dihydroethidium (DHE) staining was used to detect the expression of ROS in each tumor tissue. At the same time, each organ (heart, liver, spleen, lung, kidney) was stripped, while H&E staining was performed to detect the safety of each treatment. Mice blood was extracted for blood biochemistry and routine blood tests, which were used to characterize the safety of different treatments. Another portion of mice was used for survival study and body weight monitoring.

#### In vivo CSC proportion assay

At the endpoint of the pharmacodynamic experiment, mice were sacrificed, tumors were stripped from the mice, and clipped and ground into single-cell suspensions, and the cells were stained for CD133, CD44, CD24, and side-population cell tumor stem cell-related markers, followed by flow cytometry analysis to quantify the percentage of CSC phenotype in each tumor tissue of different treatments.

#### In vivo CSC function assay with 3D gel experiment

Mice were sacrificed at the endpoint of the pharmacodynamic experiment, and the tumors were stripped from the mice, which were sheared and ground into single-cell suspensions in a sterile ultra-clean bench. The concentration of single-cell suspensions was adjusted to 4 × 10^4^ by counting, and the concentration of fibrin mother liquor was diluted to 2 mg/ml using T7 buffer. The two solutions were mixed with a ratio of 1:1 and were added to a volume of 50 μl per well in a pre-laid 1-μl thrombin in a 96-well plate, which was then placed back into the cell incubator at 37 °C, 5% CO_2_ for 20 min to solidify. Afterward, 200 μl of DMEM medium was added and then put back into the cell incubator for incubation. The size of tumor spheroids was measured on days 1, 3, 5, and 7, and the number of tumor spheroids was counted on the seventh day [67].

#### In vivo WB experiments

Mice were sacrificed at the endpoint of the pharmacodynamic experiment, tumors were stripped from the mice and clipped, and radioimmunoprecipitation assay (RIPA) lysate was added to them and milled using a homogenizer, followed by further cell fragmentation using a cell crusher. The supernatants were centrifuged at 12000 rpm for 20 min, and the BCA kit assay was utilized to determine the amount of protein. The latter operation was the same with in vitro WB experiment.

### Real-time quantitative polymerase chain reaction experiments

Real-time quantitative polymerase chain reaction (qRT-PCR) was used to detect the RNA expression levels of HIF-1α, GLUT1, and LDHA in tumor tissues. The experiment was divided into the following steps. (a) RNA extraction: The stripped tumor tissues were cut and ground using a homogenizer, and further crushed using a cell crusher. Then, total RNA in the tumor tissues was extracted by adding Trizol. (b) Reverse transcription of RNA to cDNA: The process followed the Reverse Transcription Reaction System kit, and the specific method refers to the instructions. (c) qPCR using SYBR Green I chimeric fluorescence method: The specific method refers to the instructions. The primer sequences are as follows.

**Table:** 

Gene		Sequence
HIF-1α	F primer	ACCTTCATCGGAAACTCCAAAG
	R primer	CTGTTAGGCTGGGAAAAGTTAGG
Slc2a1	F primer	CAGTTCGGCTATAACACTGGTG
	R primer	GCCCCCGACAGAGAAGATG
LDHA	F primer	TGTCTCCAGCAAAGACTACTGT
	R primer	GACTGTACTTGACAATGTTGGGA
β-actin	F primer	GGCTGTATTCCCCTCCATCG
	R primer	CCAGTTGGTAACAATGCCATGT

#### In vivo biodistribution of CuET@PH NPs

To investigate biodistribution, CuET@PH NPs were labeled with Cy5.5. HS-HES (50 mg) reacted with Cy5.5-maleimide (1 mg) in DMSO for 12 h at 25 °C to synthesize HS-HES-Cy5.5. The product was purified, acquired with dialysis (3500 Da), and freeze-dried. CuET@PH-Cy5.5 NPs were fabricated with the preparation process described above. CuET@PH-Cy5.5 NPs were administrated to orthotopic Panc02 tumor-bearing mice with a dosage of 4 mg/kg. Meanwhile, the mixture solution of DSF, CuCl_2_, and free Cy5.5 was administrated to mice with the same dosage of CuET and Cy5.5. Three HBO treatments were given to mice before drug administration for HBO groups. PerkinElmer IVIS Lumina system was utilized to monitor drug biodistribution in mice at different time points. All mice were sacrificed, and major organs were extracted at the end of the experiments. To determine the content of drugs in tumor cells, tumor tissues were processed into single-cell suspension, and the fluorescence intensity of Cy5.5 in tumor cells was determined by flow cytometry.

### Data processing

Graphs were produced by GraphPad Prism 8.0, and graphical data were presented as mean ± SEM and analyzed for significant differences using Student’s *t* test. *P < 0.05, **P < 0.01, ***P < 0.001, ****P < 0.0001, and ns stands for not significant.

## Data Availability

The data that support the findings of this study are available from the corresponding author upon reasonable request.
