# Supplementary material for: Hyperbaric Oxygen Boosts Antitumor Efficacy of Copper-Diethyldithiocarbamate Nanoparticles against Pancreatic Ductal Adenocarcinoma by Regulating Cancer Stem Cell Metabolism
Source: Research (Wash D C). 2024 Mar 11;7:0335. doi: 10.34133/research.0335 (PMC11100349; doi:10.34133/research.0335)
Supplement: Supplementary 1 — Figs. S1 to S24 Tables S1 to S6 [file research.0335.f1.docx]

**Supplementary Information for**

**Hyperbaric oxygen boosts antitumor efficacy of hydroxyethyl starch and polydopamine stabilized copper-diethyldithiocarbamate nanoparticles against pancreatic ductal adenocarcinoma by regulating cancer stem cell metabolism**

Chen Xiao^1, #^, Jiayuan Li^1, #^, Ao Hua^1^, Xing Wang^1^, Shiyou Li^1^, Zheng Li^1^, Chen Xu^1^, Zhijie Zhang^1^, Xiangliang Yang^1, 2, 3, 4, 5, 6, *^, Zifu Li^1, 2, 3, 4, 5, 6, *^

^1^ Department of Nanomedicine and Biopharmaceuticals, College of Life Science and Technology, Huazhong University of Science and Technology, Wuhan, 430074, P. R. China

^2^ National Engineering Research Center for Nanomedicine, Huazhong University of Science and Technology, Wuhan, 430074, P. R. China

^3^ Key Laboratory of Molecular Biophysics of Ministry of Education, Huazhong University of Science and Technology, Wuhan, 430074, P. R. China

^4^ Hubei Key Laboratory of Bioinorganic Chemistry and Materia Medical, Huazhong University of Science and Technology, Wuhan, 430074, P. R. China

^5^ Hubei Engineering Research Center for Biomaterials and Medical Protective Materials, Huazhong University of Science and Technology, Wuhan, 430074, P. R. China

^6^ Hubei Bioinformatics and Molecular Imaging Key Laboratory, Huazhong University of Science and Technology, Wuhan, 430074, P. R. China

^#^ These authors contributed equally.

* Address correspondence to:

Zifu Li, Ph.D., Professor

1037 Luoyu Road, Wuhan, 430074, P. R. China

E-mail: zifuli@hust.edu.cn

Xiangliang Yang, Ph.D., Professor

1037 Luoyu Road, Wuhan, 430074, P. R. China

E-mail: yangxl@hust.edu.cn

**Supplementary Figures**


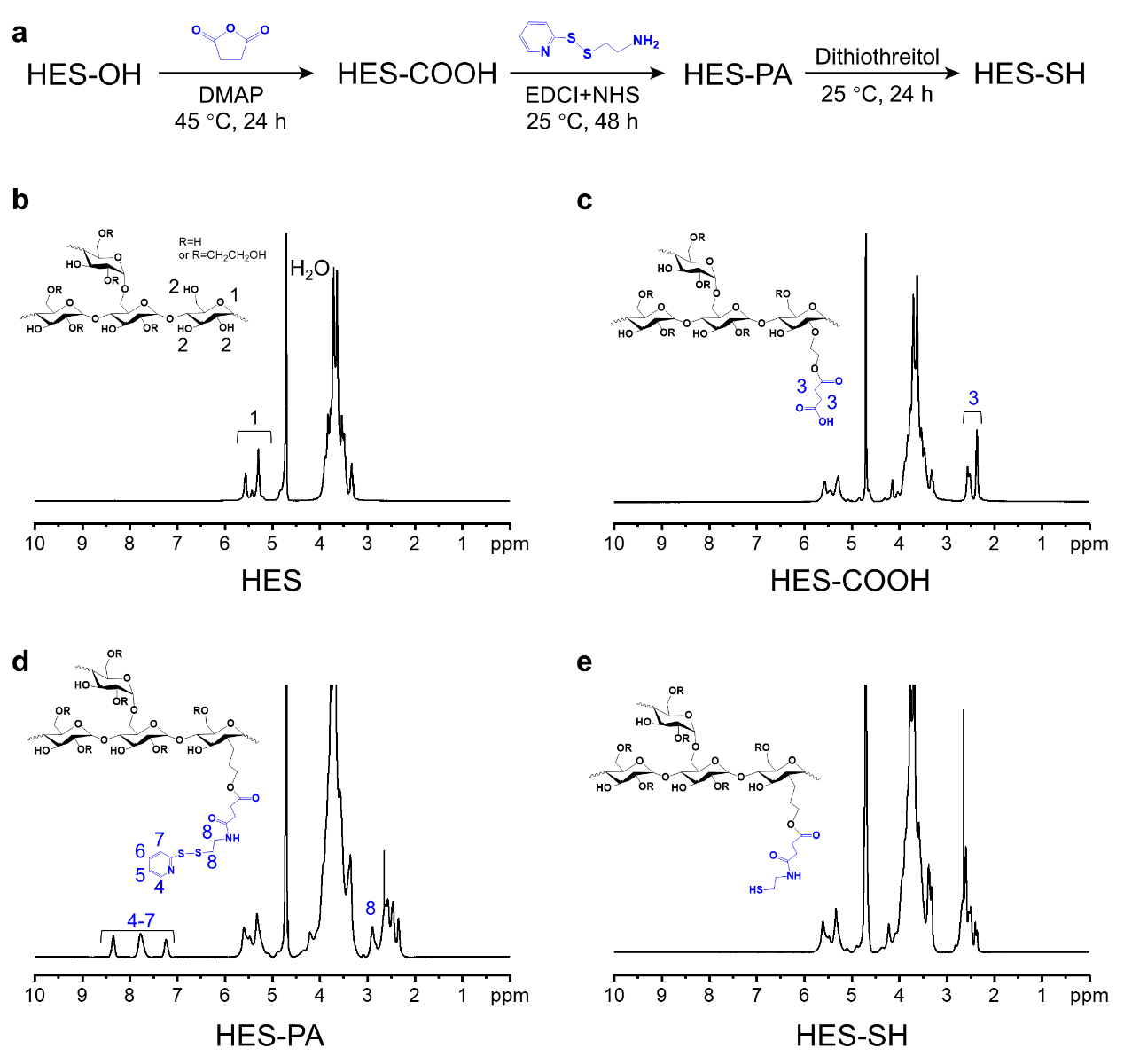


**Figure S1.** Synthesis processes of HES-SH (a) and ^1^H-NMR spectra of HES (b), HES-COOH (c), HES-PA (d) and HES-SH (e) in D_2_O.


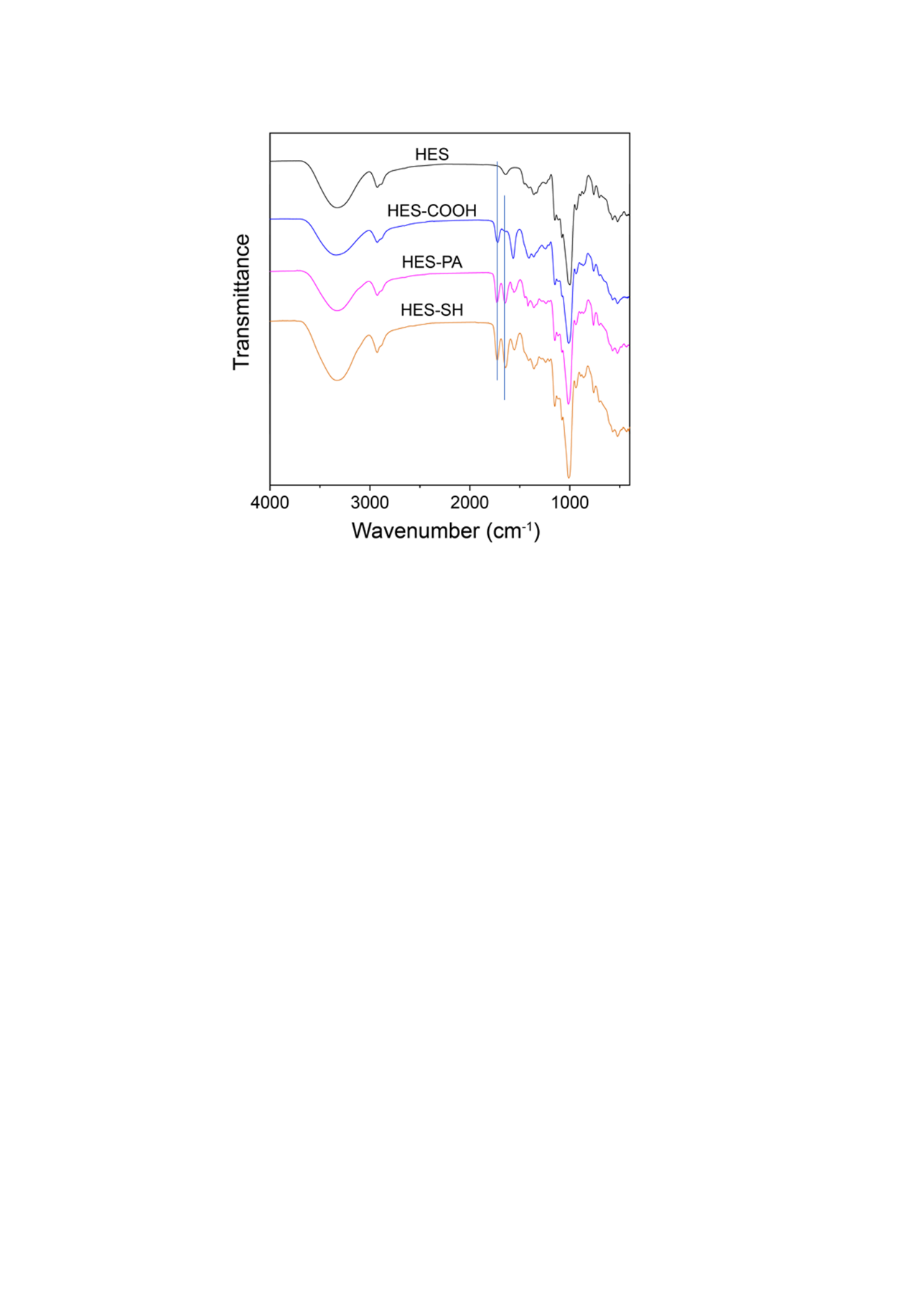


**Figure S2.** FT-IR spectra of HES, HES-COOH, HES-PA and HES-SH.


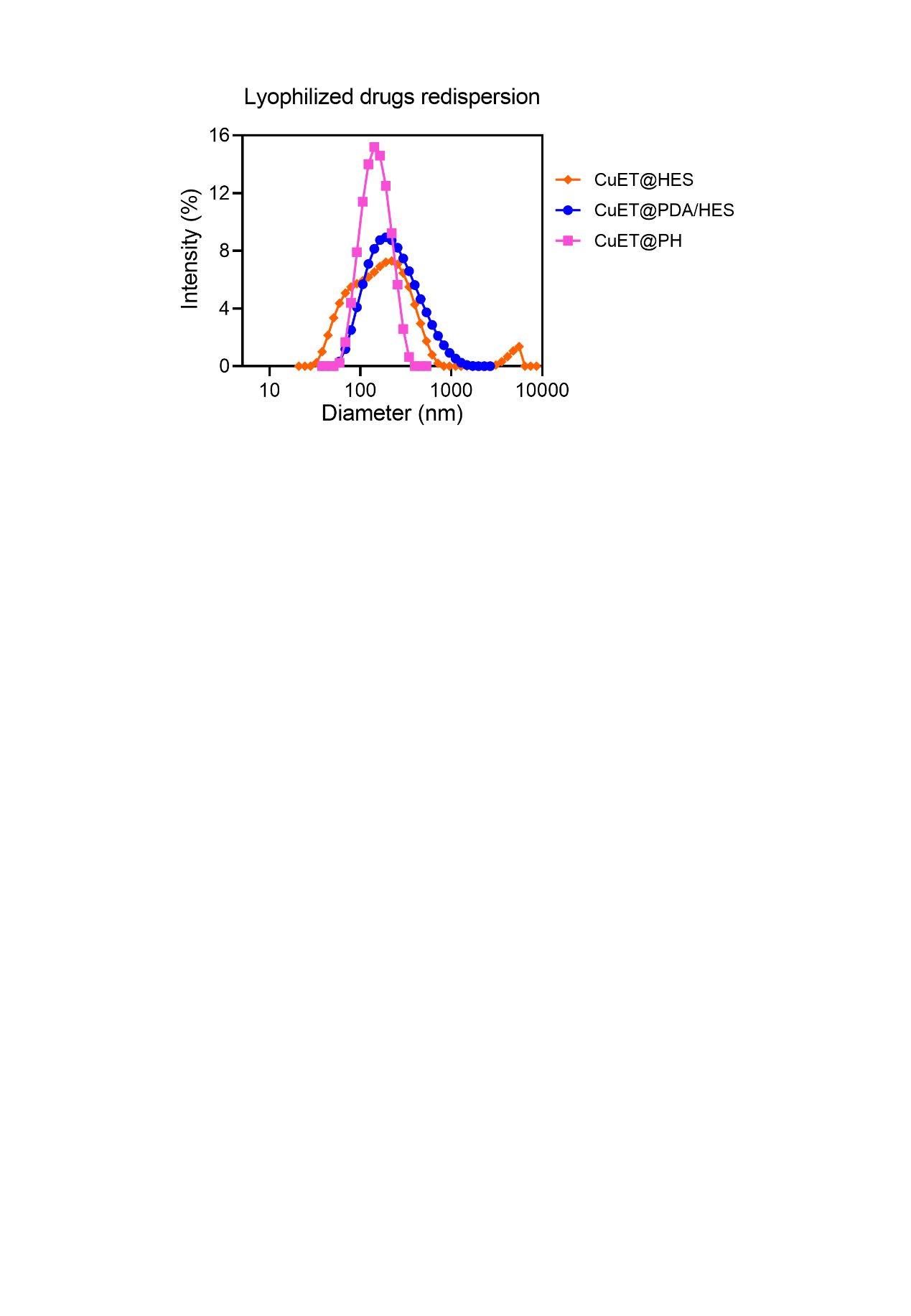


**Figure S3.** Size distributions of lyophilized CuET@HES, CuET@PDA/HES and CuET@PH NPs after redispersed in water.


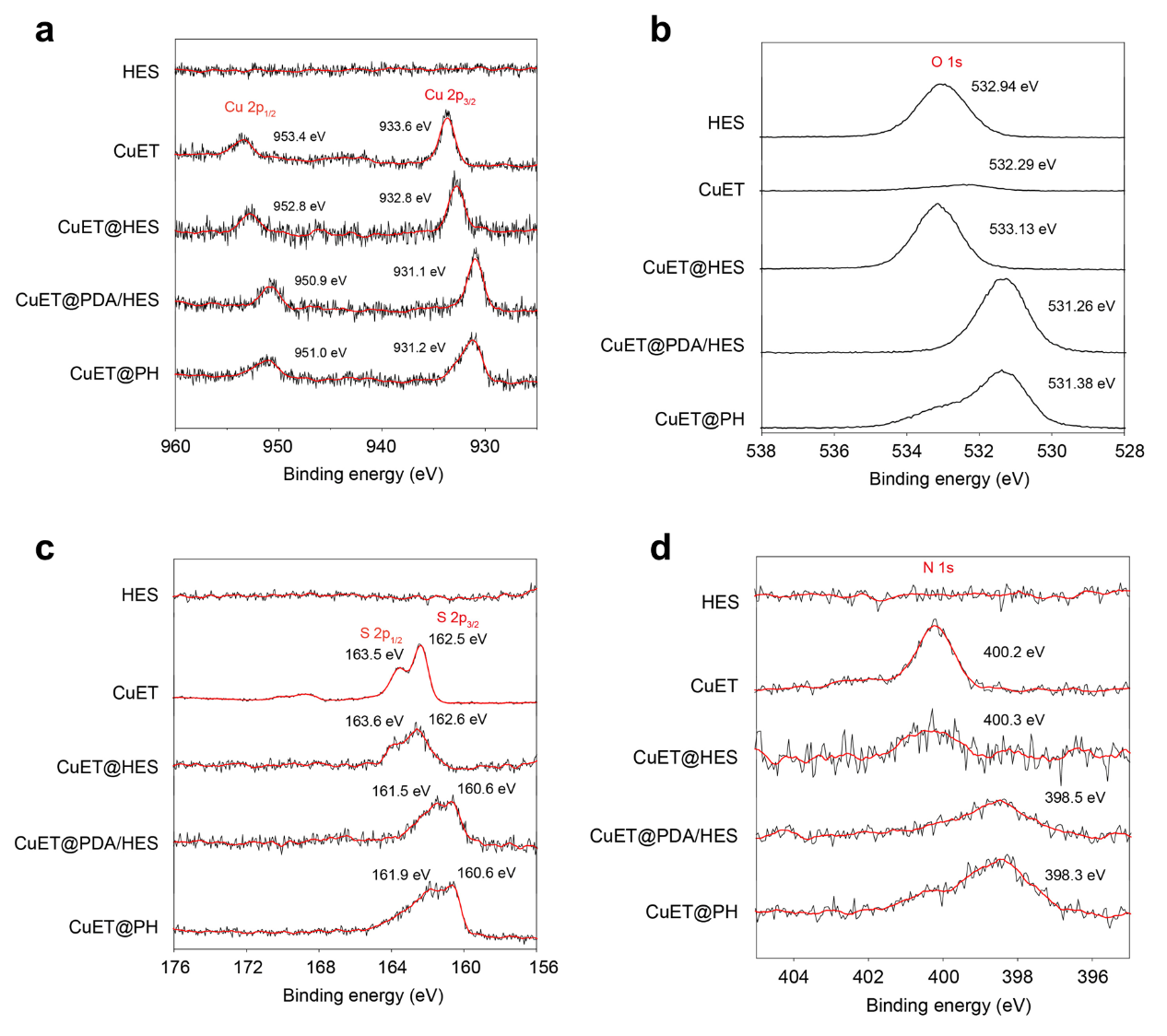


**Figure S4.** XPS spectra of CuET@PH NPs. Cu (a), O (b), S (c) and N (d) XPS spectra of HES, CuET, CuET@HES, CuET@PDA/HES and CuET@PH NPs.


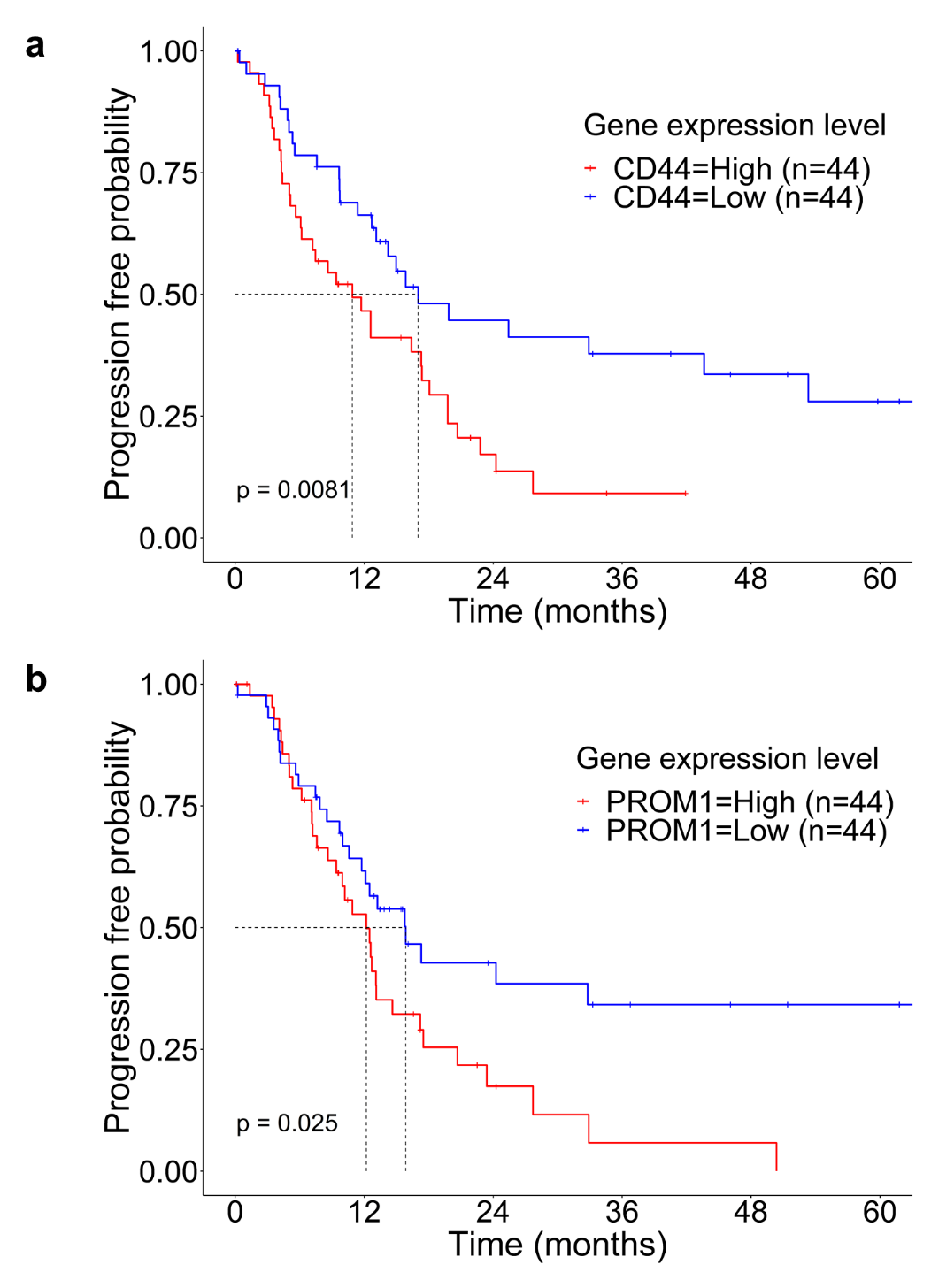


**Figure S5.** TCGA analysis. a. Relationship between progression free survival and CD44 expression in patients with pancreatic ductal adenocarcinoma. b. Relationship between progression free survival and CD133 expression in patients with pancreatic ductal adenocarcinoma.


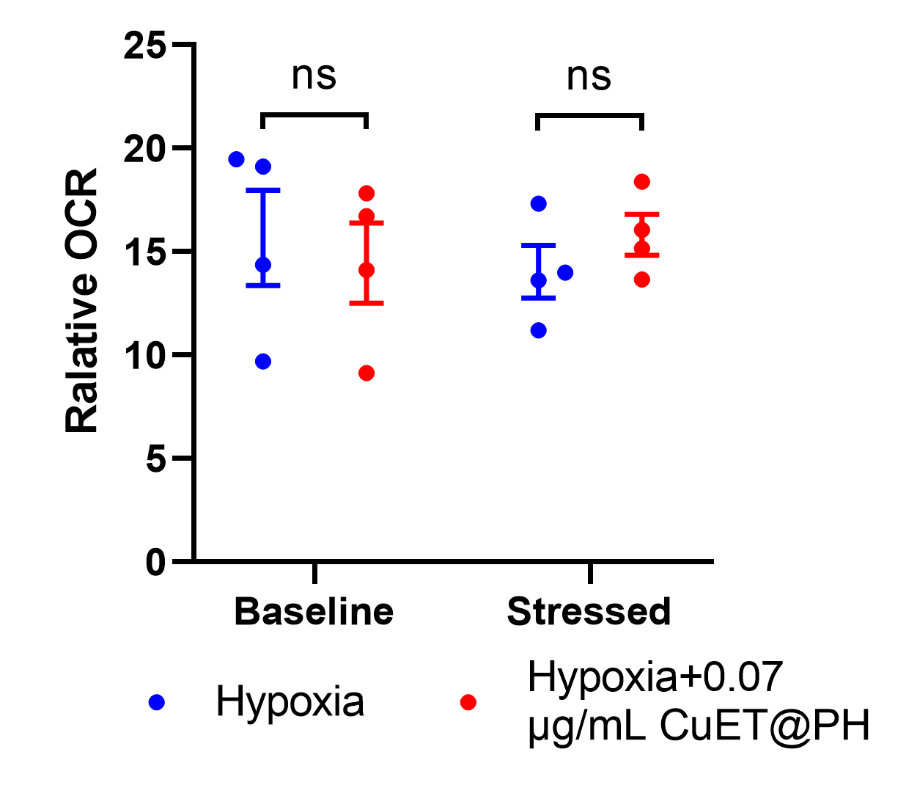


**Figure S6.** Quantitative analysis of relative OCR of hypoxia versus hypoxia combined with 0.07 μg/mL CuET@PH under baseline as well as stressed conditions (mean ± SEM, n = 4). Statistical significance was calculated by t-test. ns stands for not significant.


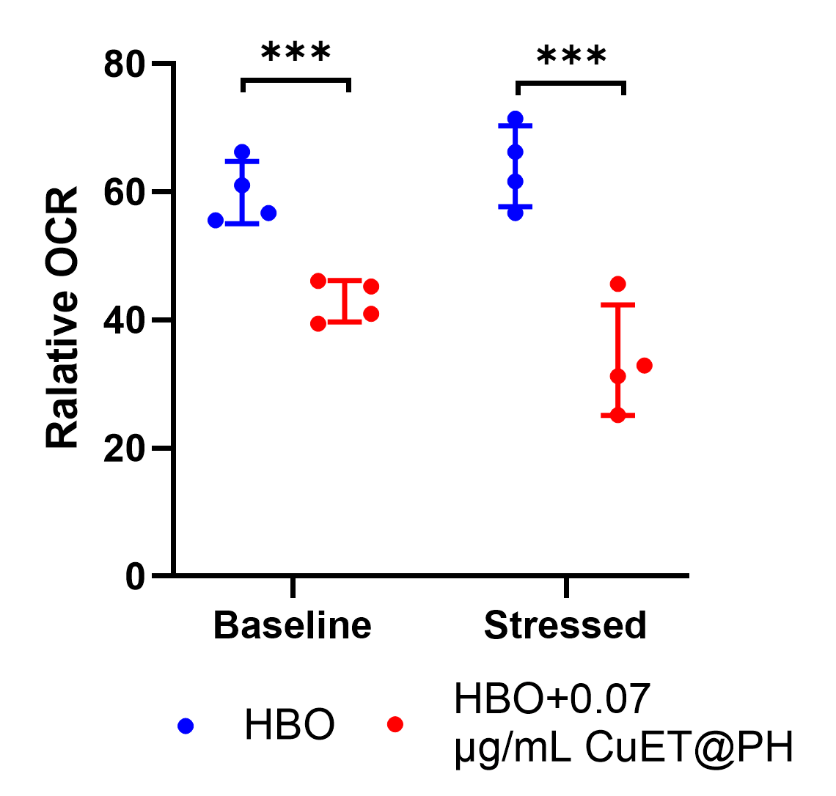


**Figure S7.** Quantitative analysis of relative OCR of HBO versus HBO combined with 0.07 μg/mL CuET@PH under baseline as well as stressed conditions (mean ± SEM, n = 4). Statistical significance was calculated by t-test. *p* values: *** *p* < 0.001.


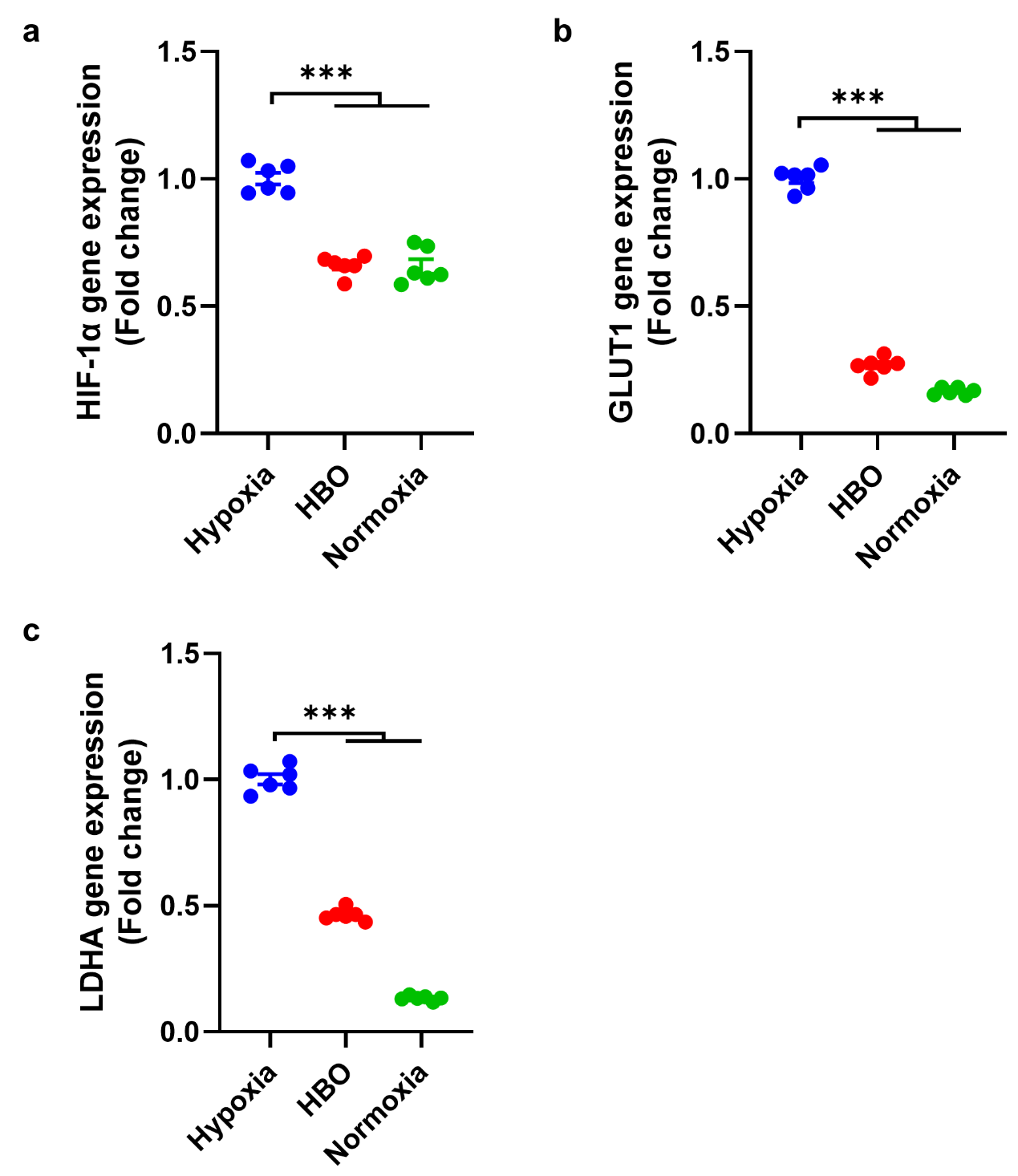


**Figure S8.** qRT-PCR quantification of HIF-1α (a), GLUT1 (b) and LDHA (c) gene expression in Panc02 CSCs after different treatments (hypoxia, HBO and normoxia) (mean ± SEM, n = 6). Statistical significance was calculated by t-test. *p* values: *** *p* < 0.001.

**
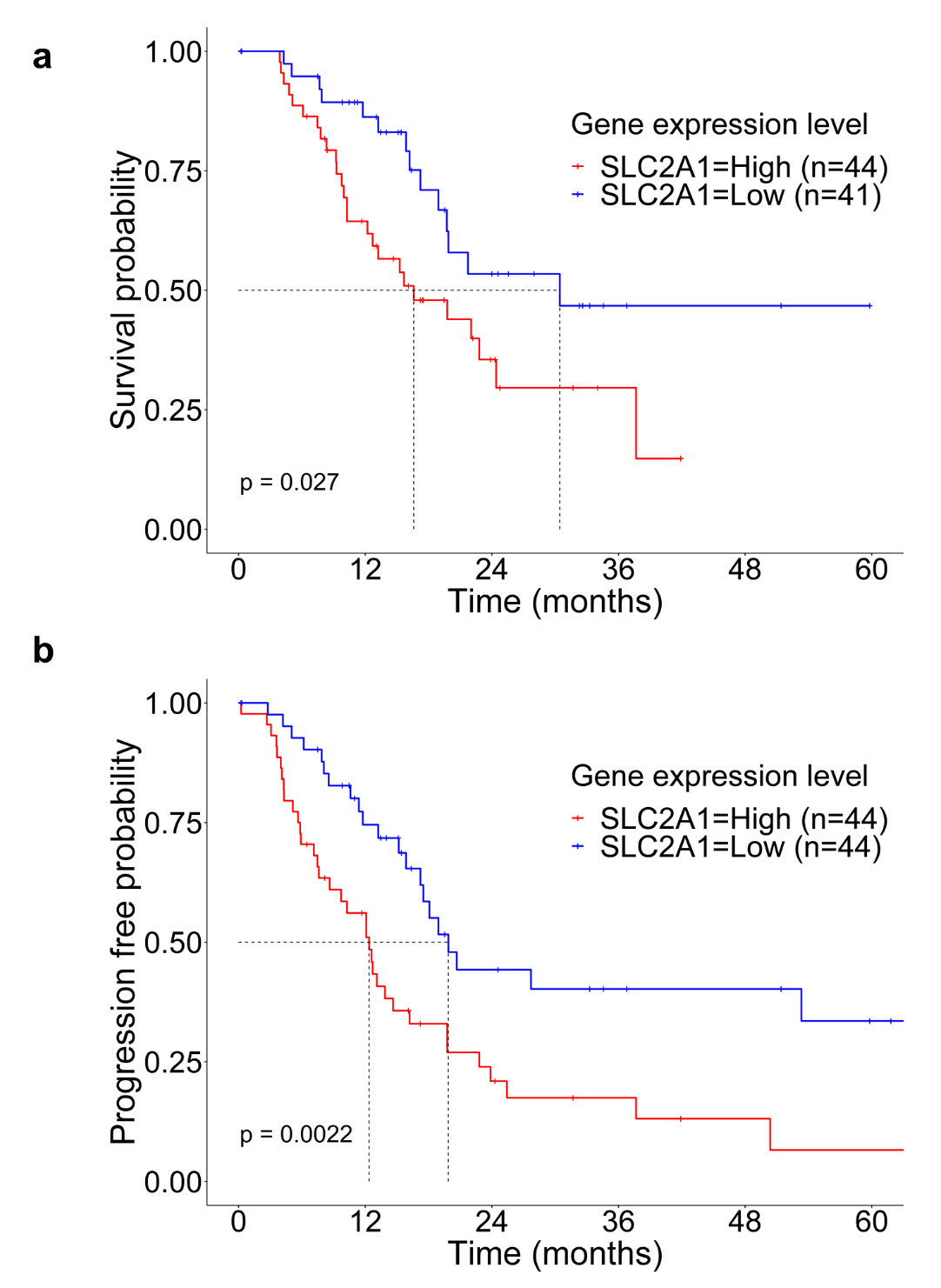
**

**Figure S9.** TCGA analysis. a. Relationship between survival probability and GLUT1 expression in patients with pancreatic ductal adenocarcinoma. b. Relationship between progression free survival and GLUT1 expression in patients with pancreatic ductal adenocarcinoma.

**
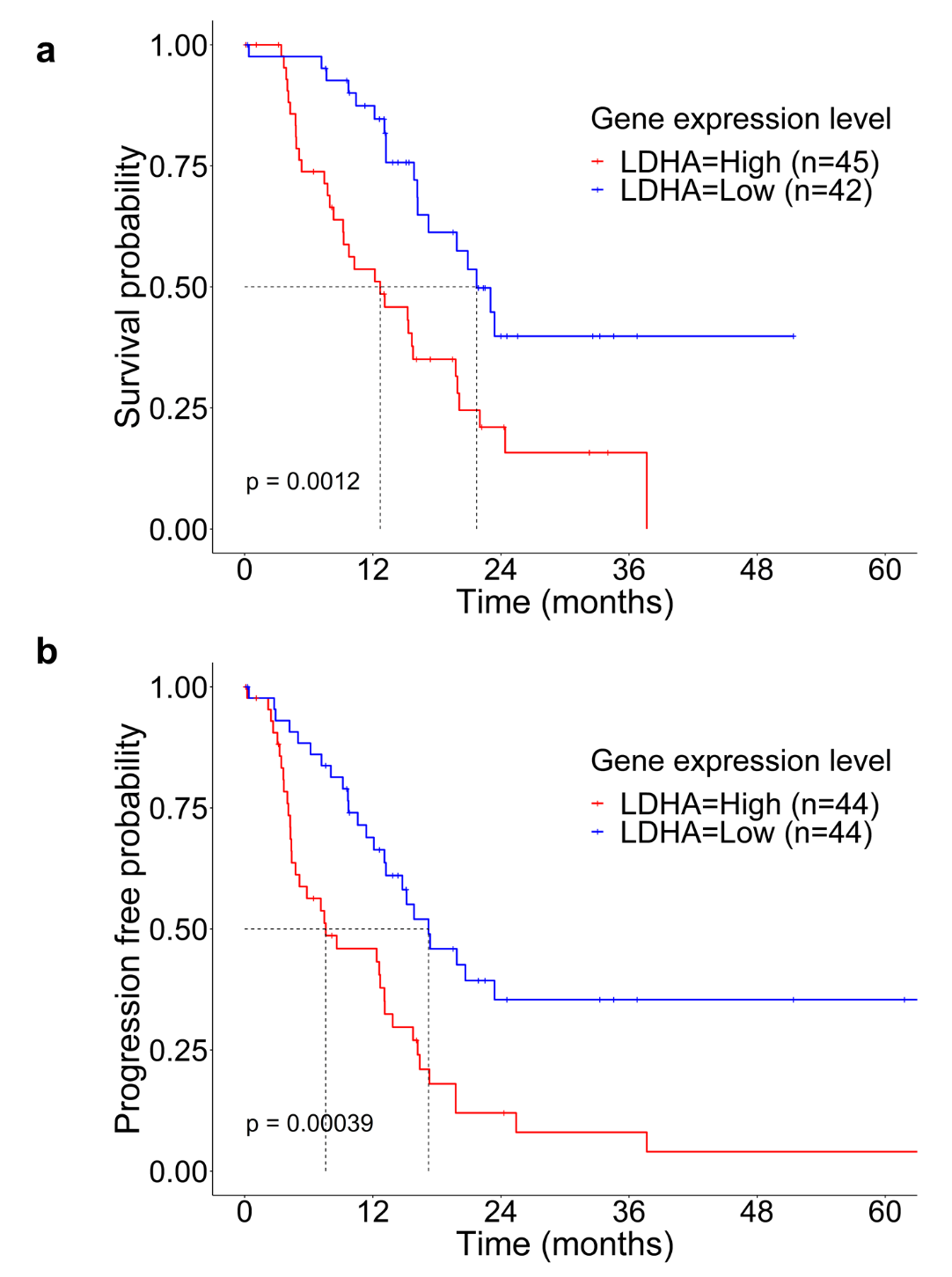
**

**Figure S10.** TCGA analysis. a. Relationship between survival probability and LDHA expression in patients with pancreatic ductal adenocarcinoma. b. Relationship between progression free survival and LDHA expression in patients with pancreatic ductal adenocarcinoma.


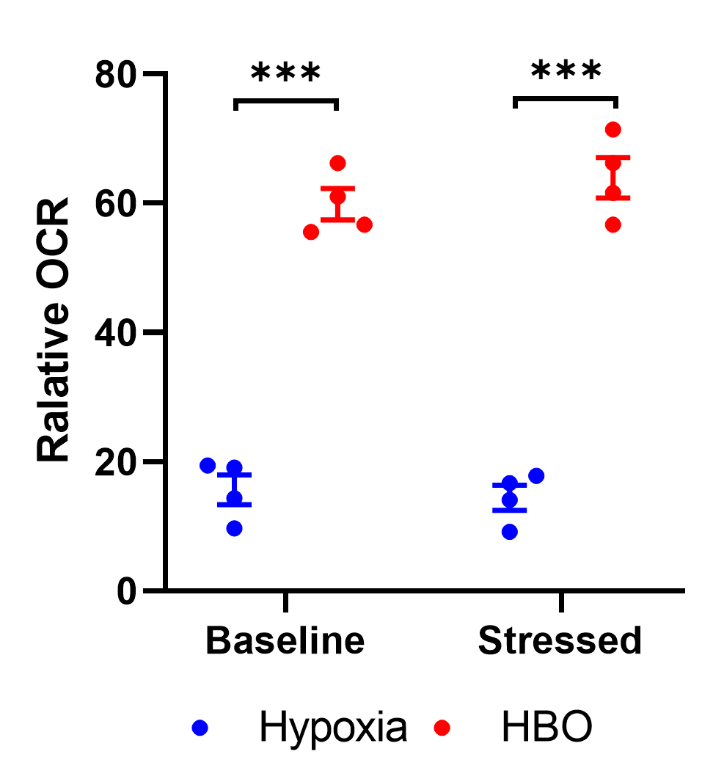


**Figure S11.** Quantitative analysis of relative OCR of hypoxia versus HBO under baseline and stressed conditions (mean ± SEM, n = 4). Statistical significance was calculated by t-test. *p* values: *** *p* < 0.001.


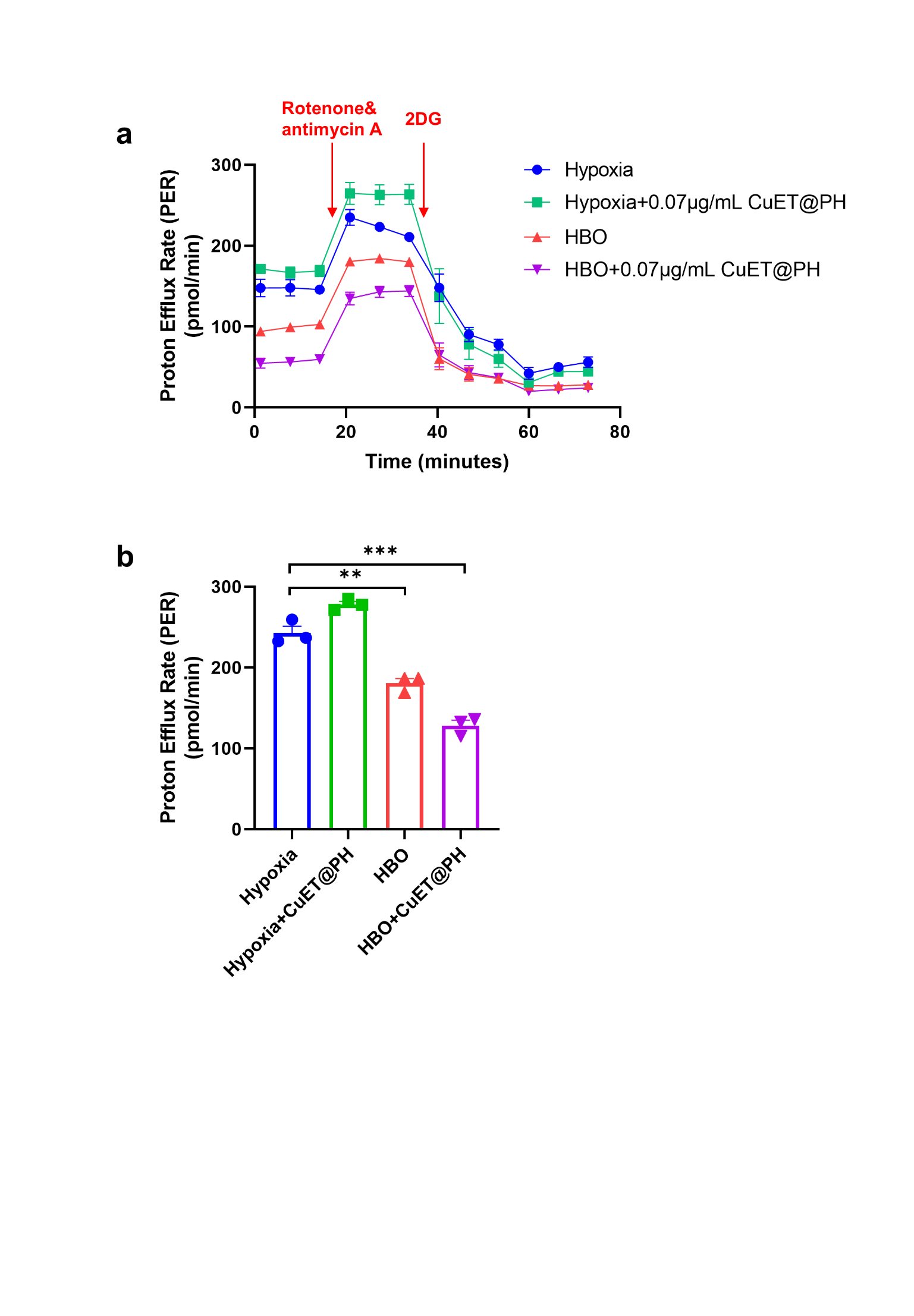


**Figure S12.** The Proton Efflux Rate (PER) of Panc02 CSCs under hypoxia, HBO, hypoxia, and HBO after 24 hours of 0.07 μg/mL CuET@PH treatment (2DG: 2-Deoxy-D-Glucose) (mean ± SEM, n = 3).


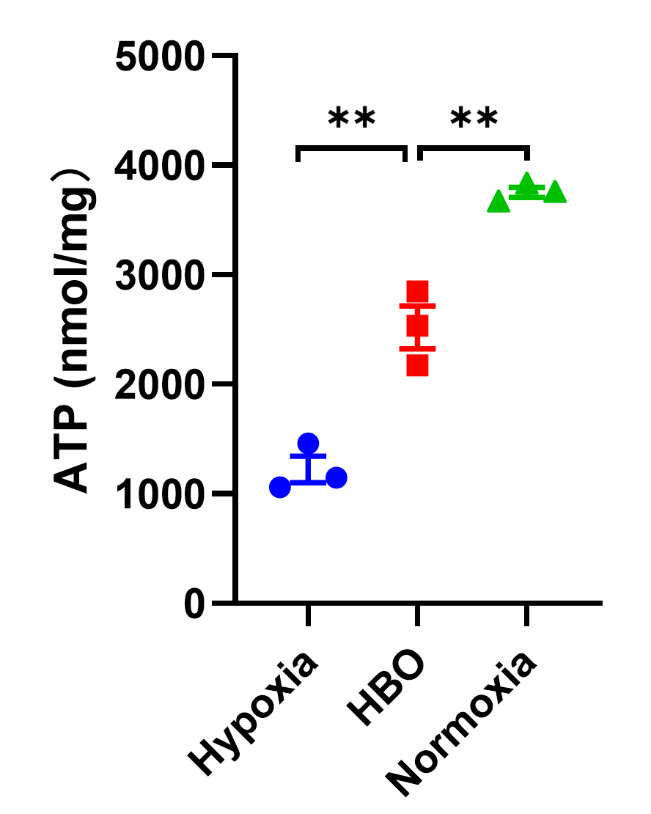


**Figure S13.** Levels of intracellular ATP under different treatments (mean ± SEM, n=3). Statistical significance was calculated by t-test. *p* values: ** *p* < 0.01.


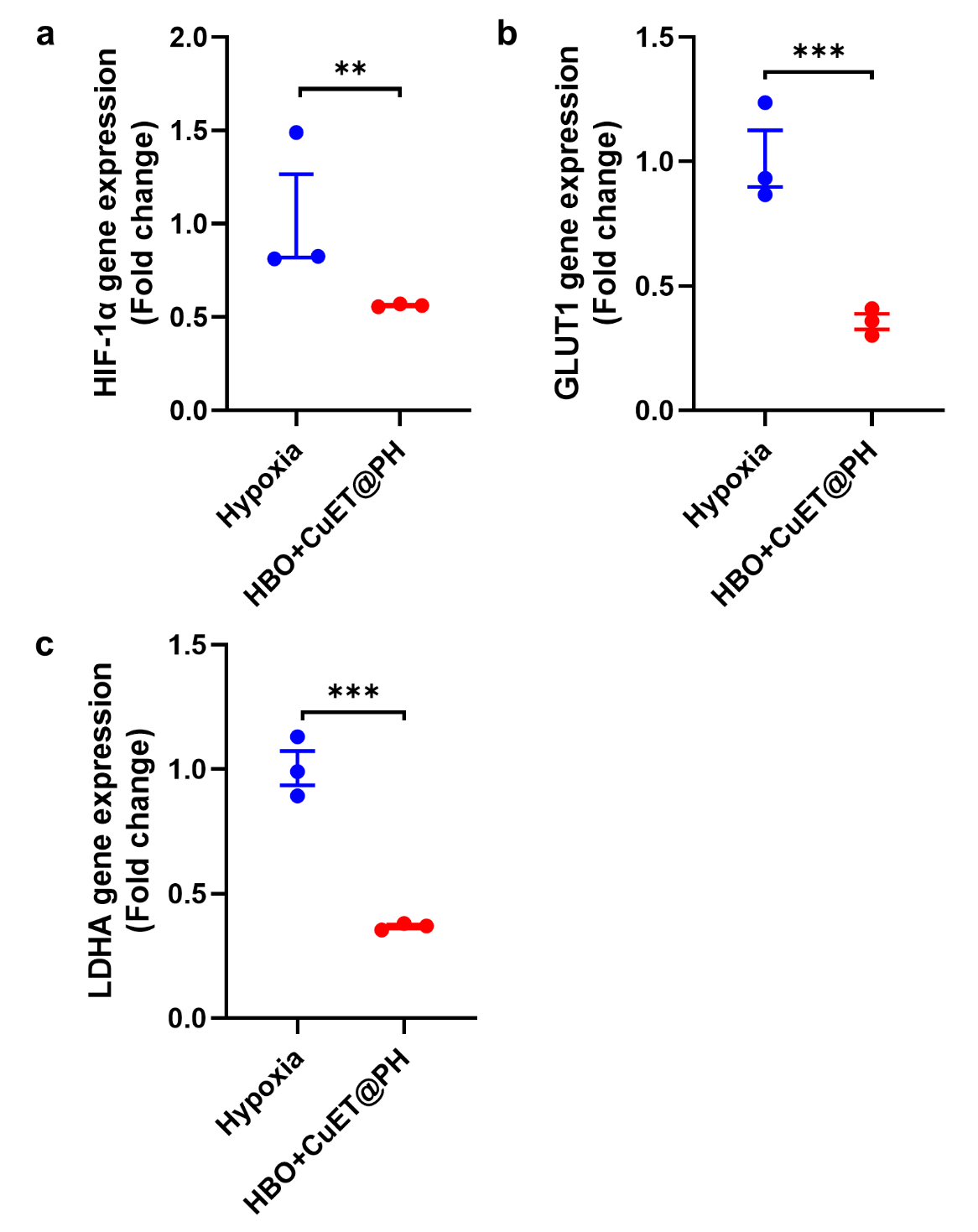


**Figure S14.** Changes of RNA levels between HBO combined with 0.05 μg/mL CuET@PH and hypoxia. qRT-PCR quantification of HIF-1α (a), GLUT1 (b) and LDHA (c) gene expression in Panc02 CSCs after different treatments (hypoxia, HBO+CuET@PH) (mean ± SEM, n = 3). Statistical significance was calculated by t-test. *p* values: ** *p* < 0.01, *** *p* < 0.001.


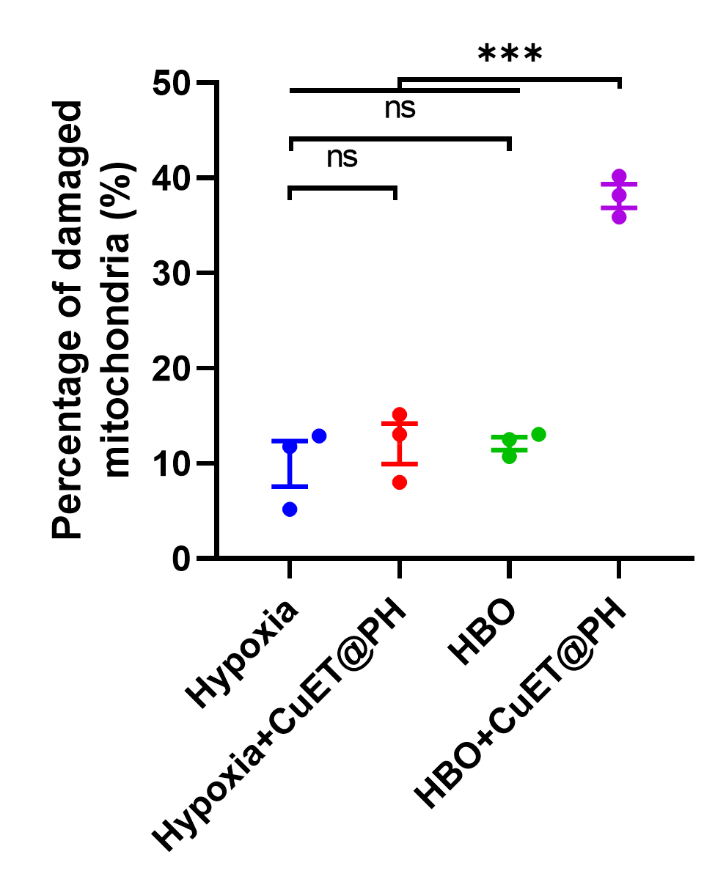


**Figure S15.** Quantification of damaged mitochondria by Bio-TEM. The concentration of CuET@PH NPs was 0.05 μg/mL (mean ± SEM, n = 3). Statistical significance was calculated by t-test. *p* values: *** *p* < 0.001; ns stands for not significant.


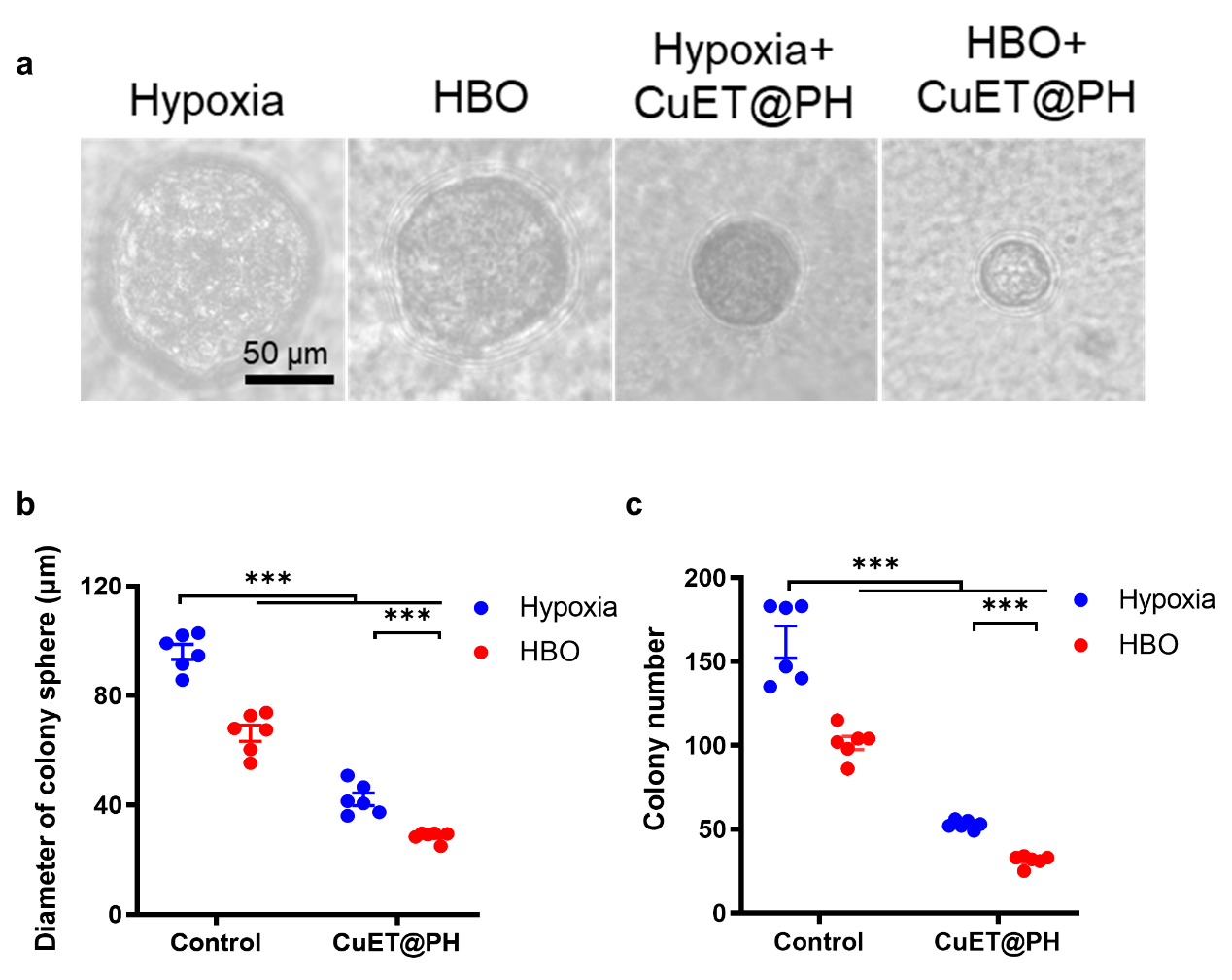


**Figure S16.** *In vitro* CSCs function assay in 3D fibrin gels. The concentration of CuET@PH NPs was 0.05 μg/mL. a. 3D fibrin gel experiments with Panc02 cancer cells from different treatments. b. Diameter of colony sphere on day 7. c. Colony number on day 7 (mean ± SEM, n = 6). Statistical significance was calculated by t-test. *p* values: *** *p* < 0.001.


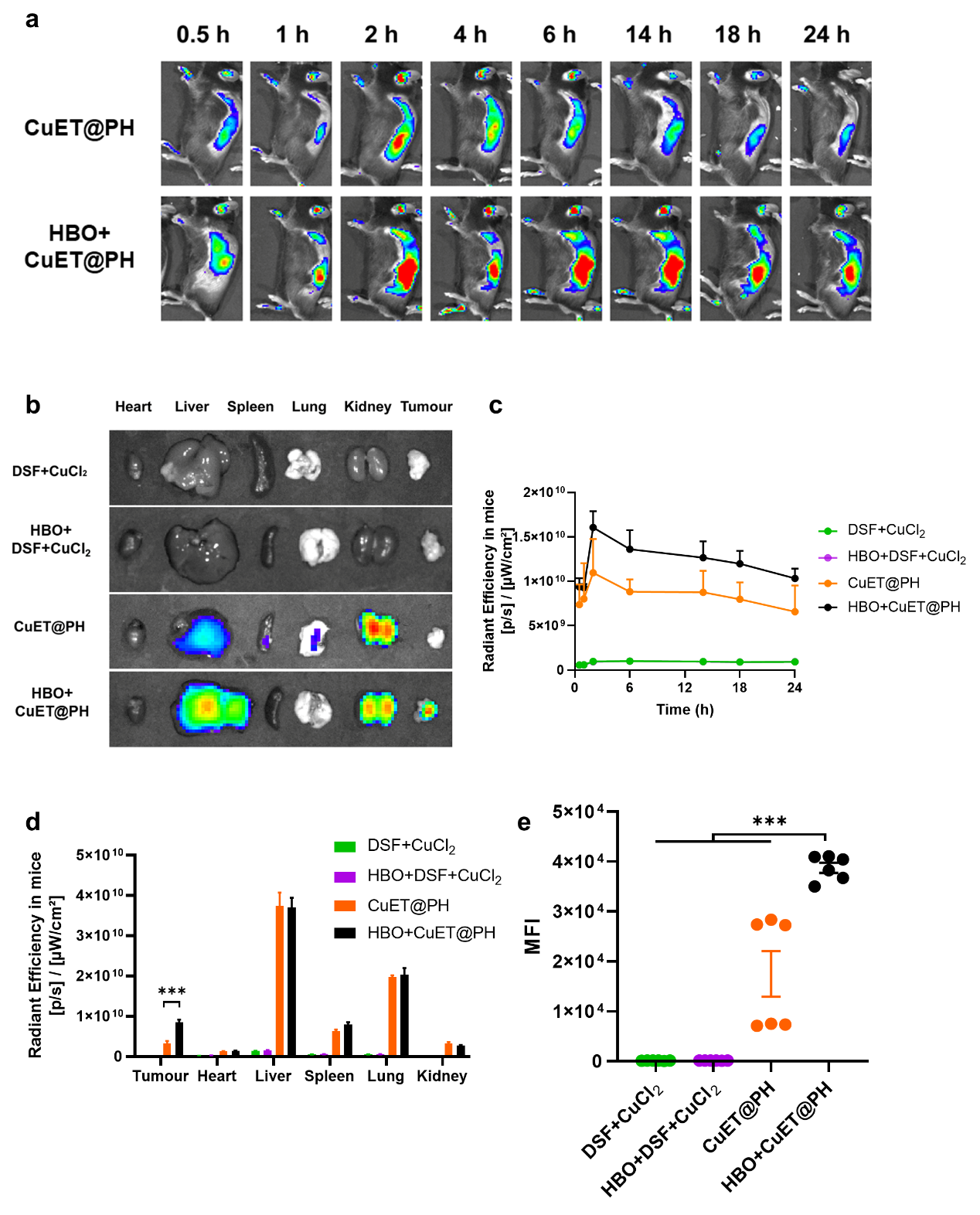


**Figure S17.** HBO enhances CuET@PH NPs tumor targeting delivery. a. *In vivo* imaging of PDAC mice. b. The fluorescence image of excised organs and tumors from mice 24h after different treatments. c. Tumor fluorescence intensity-time curve (mean ± SEM, n = 4). d. Quantitative fluorescence results for excised organs and tumors 24 h after different treatments (mean ± SEM, n = 6). e. Quantification of fluorescence intensity of tumor cells 24 h after different treatments by flow cytometry (mean ± SEM, n = 6). Statistical significance was calculated by t-test. *p* values: *** *p* < 0.001.


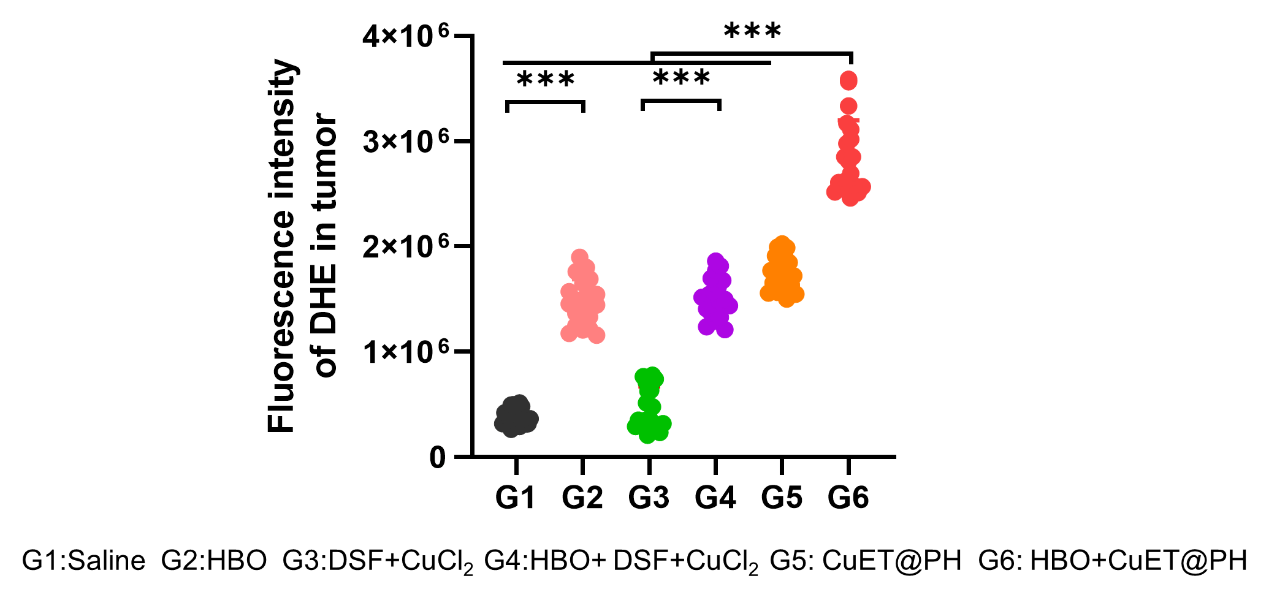


**Figure S18.** Fluorescence intensity of DHE in tumor tissues of different groups (mean ± SEM, n = 20). Statistical significance was calculated by t-test. *p* values: *** *p* < 0.001.


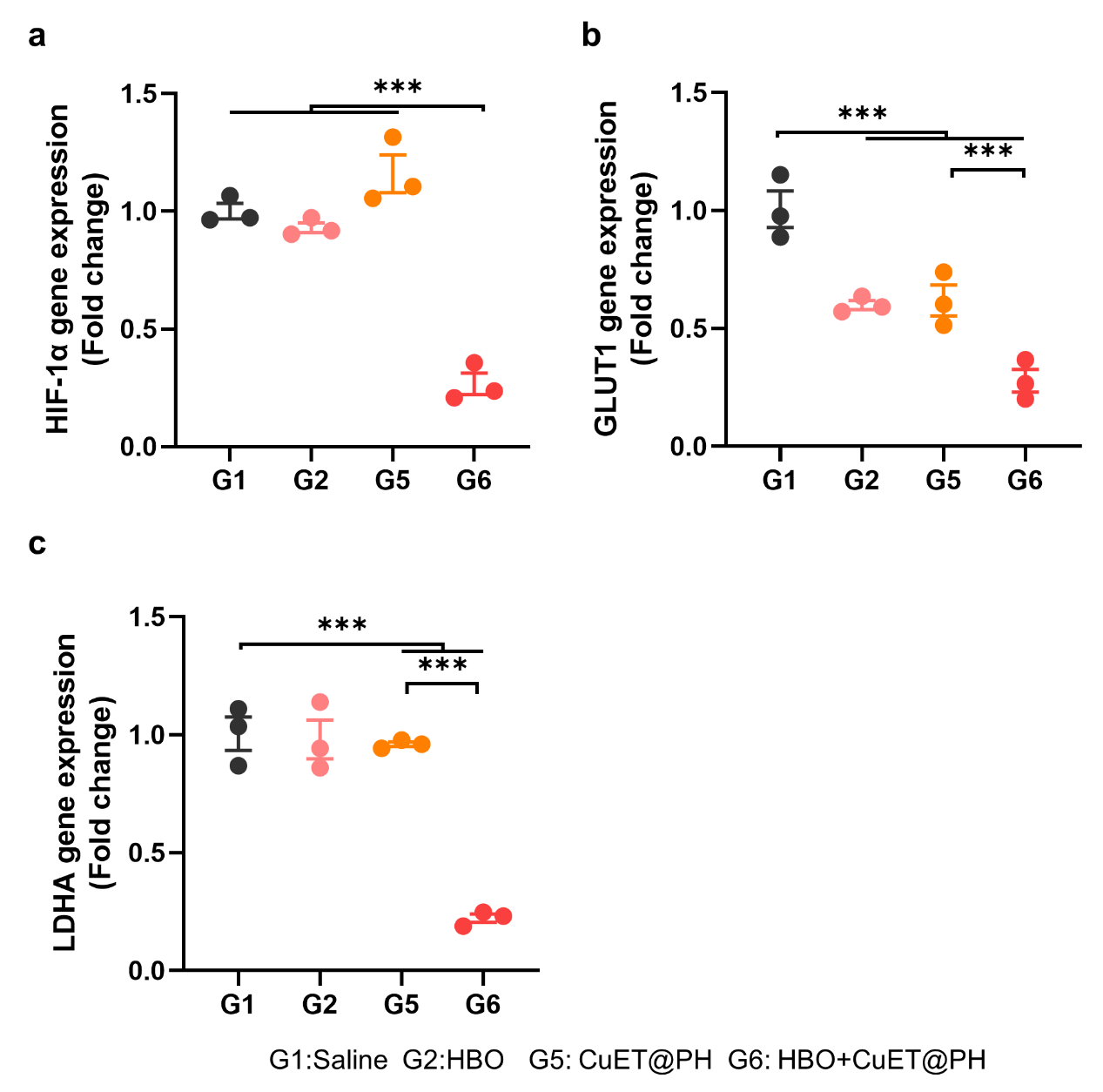


**Figure S19.** qRT-PCR quantification of gene expressions of HIF-1α (a), GLUT1 (b) and LDHA (c) in tumor tissues of different groups (mean ± SEM, n = 3). Statistical significance was calculated by t-test. *p* values: ** *p* < 0.01, *** *p* < 0.001.


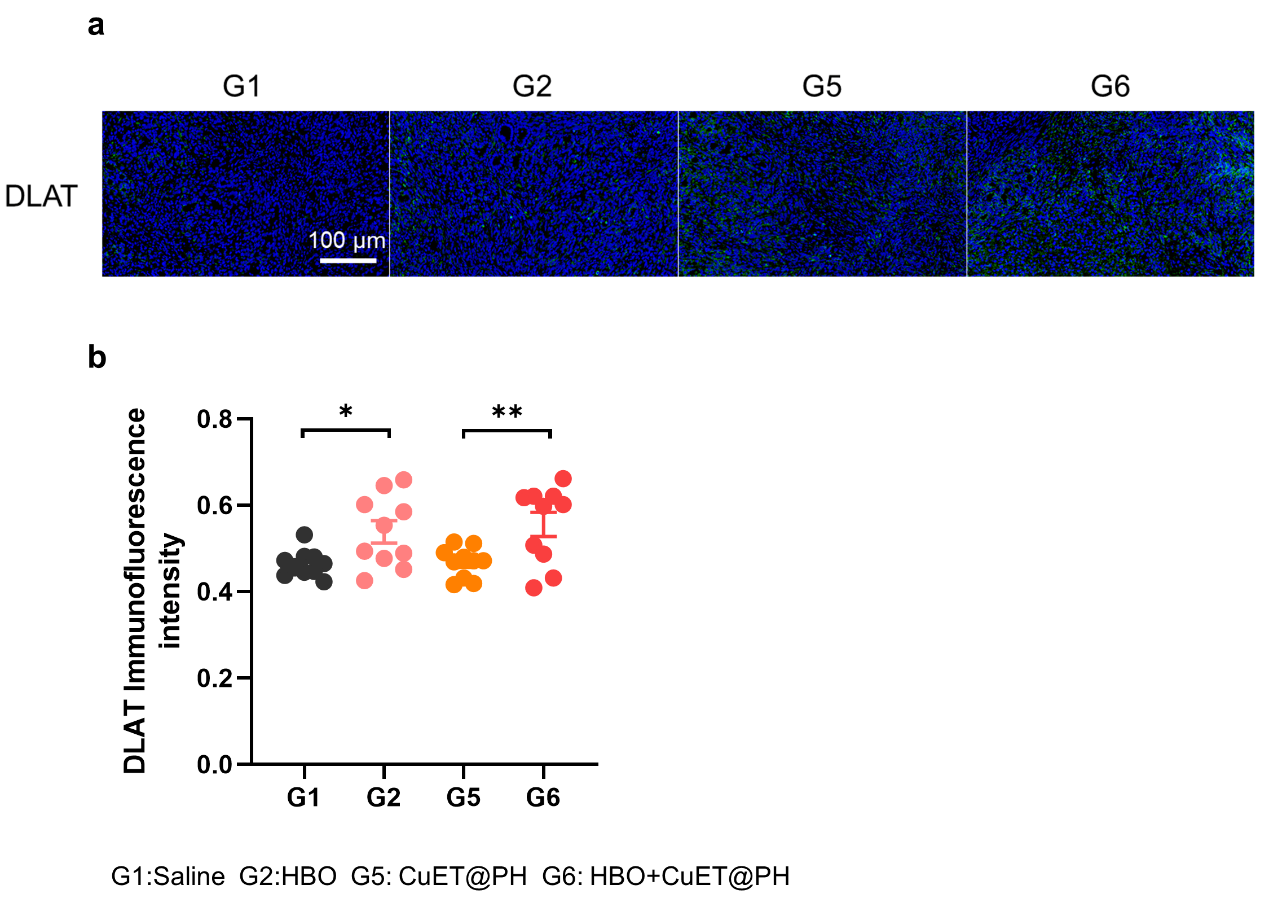


**Figure S20.** The immunofluorescence staining of DLAT (a) and semi-quantification immunofluorescence intensity of DLAT (b) in tumor tissues of different groups (mean ± SEM, n = 10). Statistical significance was calculated by t-test. *p* values: * *p* < 0.05, ** *p* < 0.01.


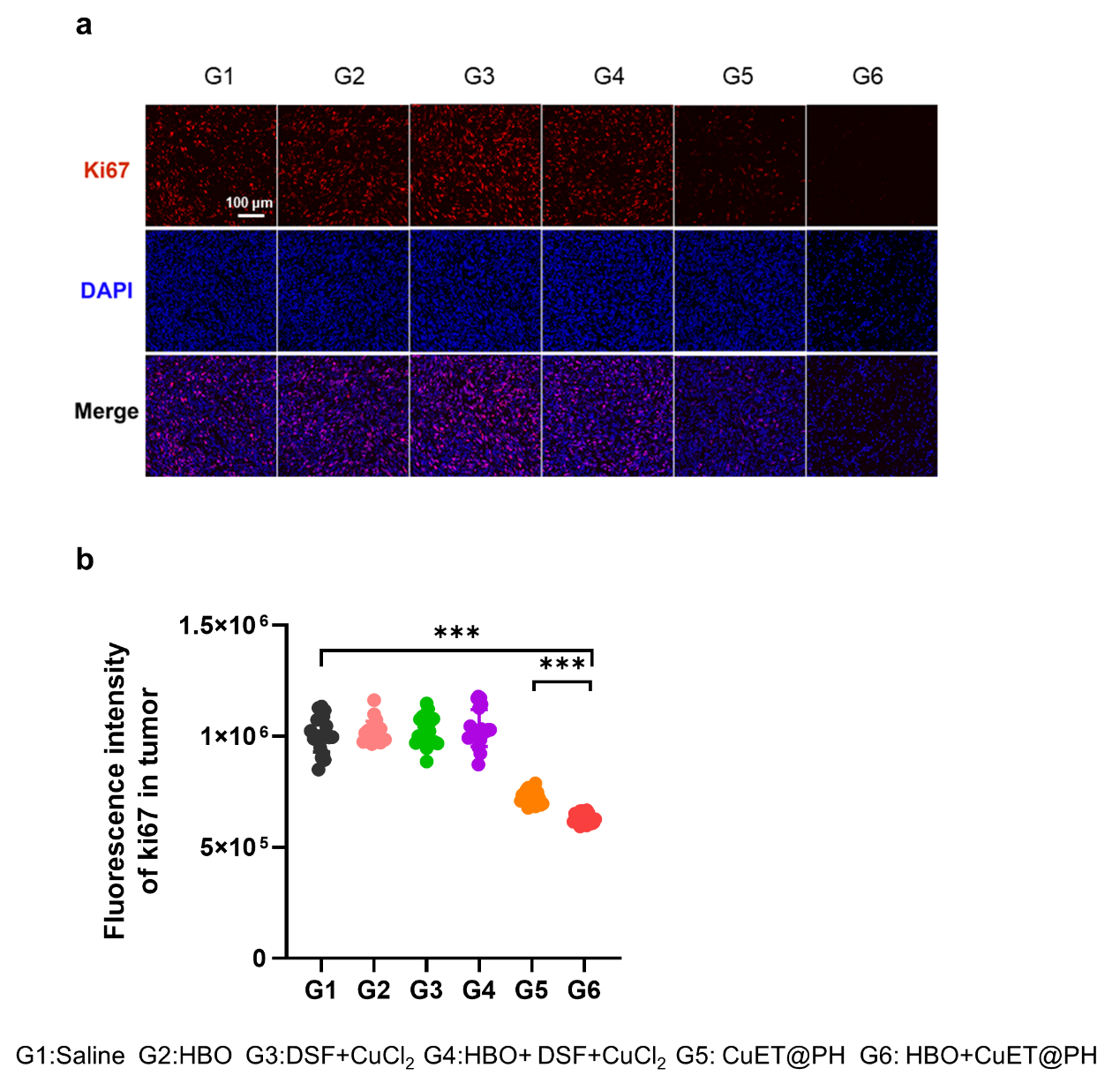


**Figure S21.** Evaluation of proliferation post various treatments. The immunofluorescence staining of ki67 (a) and semi-quantification immunofluorescence intensity of ki67 (b) in tumor tissues of different groups (mean ± SEM, n = 20). Statistical significance was calculated by t-test. *p* values: *** *p* < 0.001.


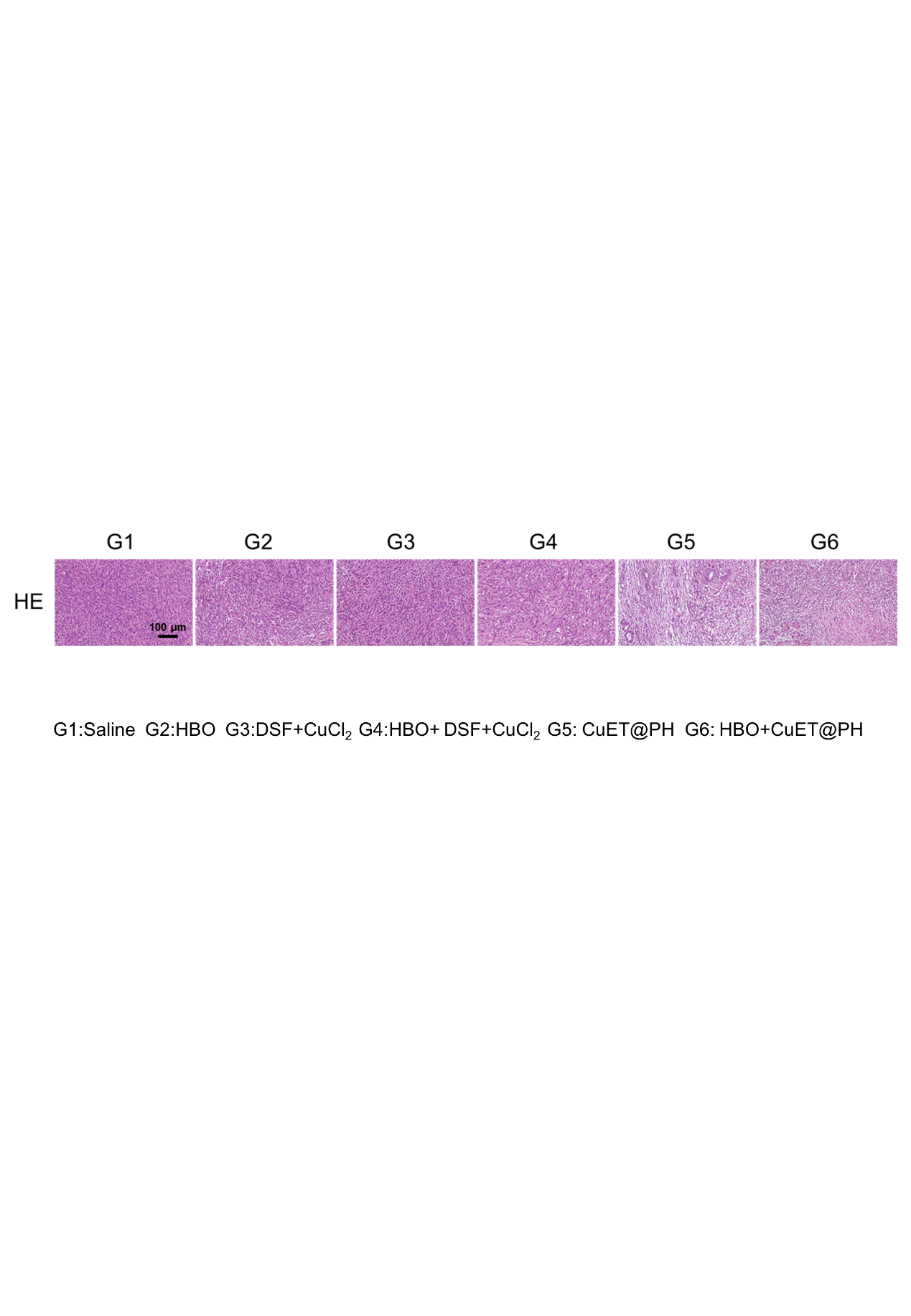


**Figure S22.** Hematoxylin eosin-stained sections of tumor tissues.


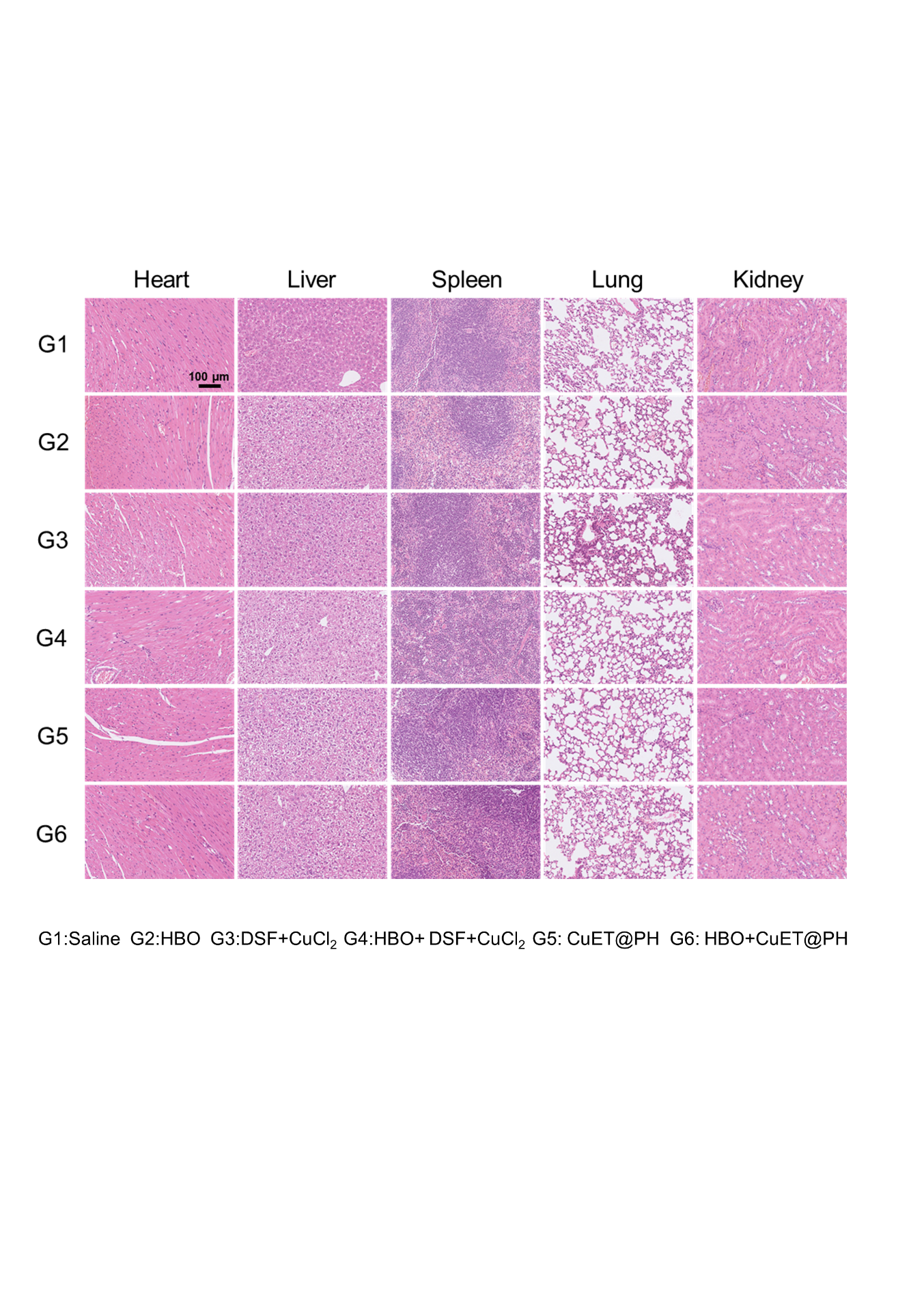


**Figure S23.** Hematoxylin eosin-stained sections of heart, liver, spleen, lung and kidney.


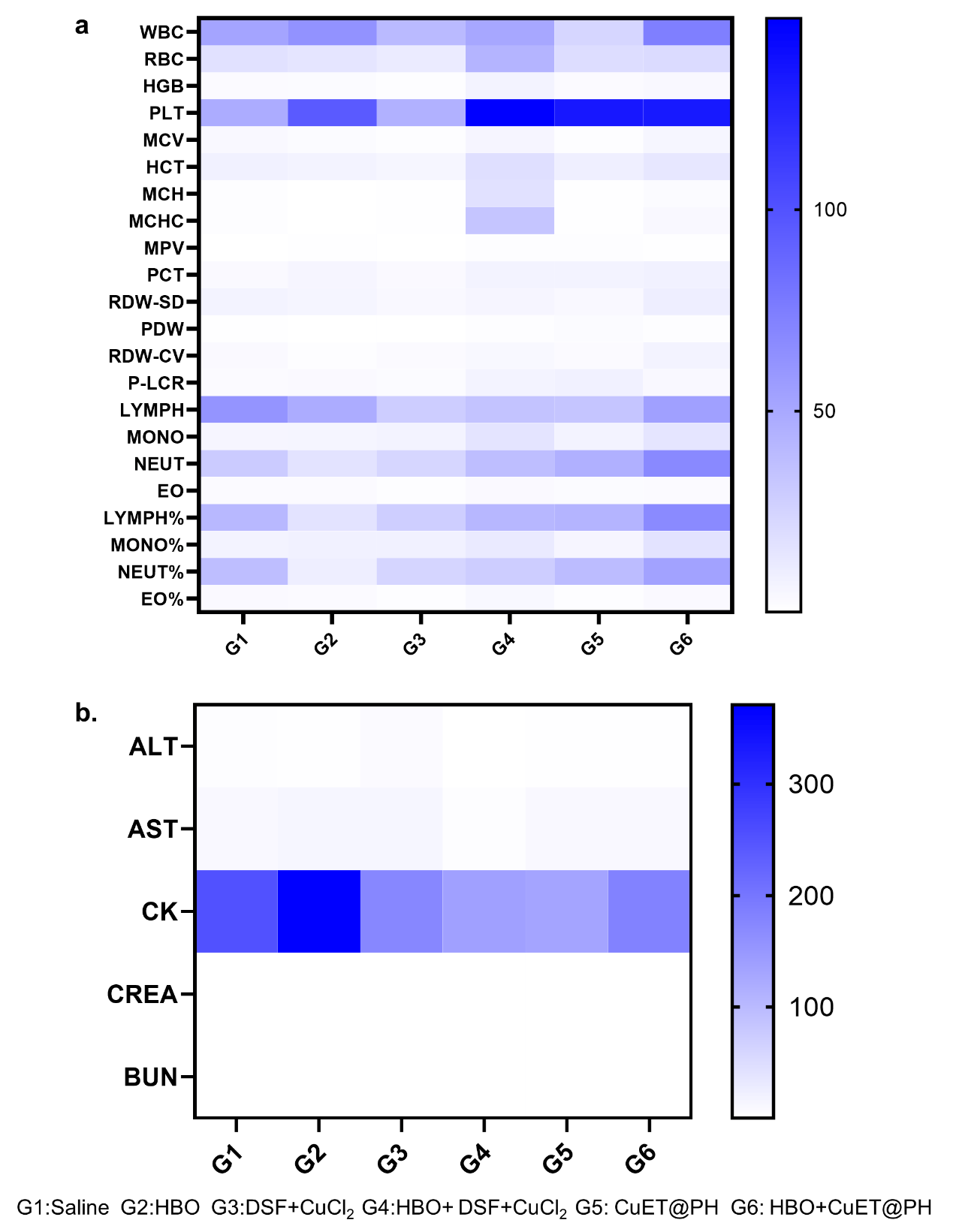


**Figure S24.** Treatment safety assay. (a) mice blood routine (WBC, RBC, HGB) and (b) blood biochemistry (ALT, BUN, CREA) data were analyzed.

**Supplementary Tables**

**Table S1.** IC50 of CuET@PH against Panc02 cancer cells in various conditions.

|  | Hypoxia | HBO | Normoxia |
| --- | --- | --- | --- |
| IC50 (μg/mL) | 0.299 | 0.134 | 0.077 |

**Table S2.** IC50 of CuET@PH against Panc02 CSCs in various conditions.

|  | Hypoxia | HBO | Normoxia |
| --- | --- | --- | --- |
| IC50 (μg/mL) | 0.086 | 0.064 | 0.062 |

**Table S3.** Up and down regulation of metabolites of hypoxia versus hypoxia combined with 0.03 μg/mL CuET@PH NPs based on targeted metabolomics analysis.

| Compounds | VIP | Fold Change | Type |
| --- | --- | --- | --- |
| BPG | 1.8651 | 3.2401 | up |
| Ornithine | 1.4554 | 2.9645 | up |
| L-Cystine | 1.2081 | 2.1722 | up |
| Isocitric-acid | 2.2339 | 0.0155 | down |

**Table S4.** Up and down regulation of metabolites of HBO versus HBO combined with 0.03 μg/mL CuET@PH NPs based on targeted metabolomics analysis.

| Compounds | VIP | Fold Change | Type |
| --- | --- | --- | --- |
| Citric-acid | 3.3460 | 206.3007 | up |
| Arginino-succinic-acid | 1.5288 | 7.1152 | up |
| Isocitric-acid | 2.3183 | 5.2942 | up |
| Itaconic-acid | 2.0391 | 0.3022 | down |
| 3-phenyllactic-acid | 1.1156 | 0.4358 | down |

**Table S5.** Up and down regulation of metabolites of hypoxia versus HBO based on targeted metabolomics analysis.

| Compounds | VIP | Fold Change | Type |
| --- | --- | --- | --- |
| Ornithine | 1.9397 | 56.1953 | up |
| Inosine | 1.1494 | 2.0596 | up |
| D (+)-Glucose | 1.0574 | 0.0666 | down |
| 6-Phospho-gluconic-acid | 1.4128 | 0.3699 | down |
| Trehalose-6-phosphate | 1.1931 | 0.4225 | down |
| 2-Phospho-D-glyceric acid | 1.3079 | 0.4806 | down |
| Phosphoenol  -pyruvic-acid | 1.4107 | 0.3036 | down |
| 3-phospho-glycerate | 1.4288 | 0.4402 | down |
| L-Cystine | 1.1665 | 0.1708 | down |

**Table S6.** Up and down regulation of metabolites of hypoxia versus HBO combined with 0.03 μg/mL CuET@PH NPs based on targeted metabolomics analysis.

| Compounds | VIP | Fold Change | Type |
| --- | --- | --- | --- |
| Citric-acid | 1.7608 | 206.3007 | up |
| Ornithine | 1.2157 | 28.9040 | up |
| L-Cystine | 1.1042 | 0.2277 | down |
| D-Erythrose-4-phosphate | 1.7390 | 0.4852 | down |
| 6-Phospho-gluconic-acid | 1.1789 | 0.4142 | down |
| Trehalose-6-phosphate | 1.2215 | 0.3842 | down |
| Phosphoenol-pyruvic-acid | 1.3578 | 0.4081 | down |
